# Biased belief priors versus biased belief updating: Differential correlates of depression and anxiety

**DOI:** 10.1371/journal.pcbi.1010176

**Published:** 2022-08-15

**Authors:** Christopher Gagne, Sharon Agai, Christian Ramiro, Peter Dayan, Sonia Bishop

**Affiliations:** 1 Department of Psychology, UC Berkeley, Berkeley, California, United States of America; 2 Max Planck Institute for Biological Cybernetics, Tübingen, Germany; 3 University of Tübingen, Tübingen, Germany; 4 Helen Wills Neuroscience Institute, UC Berkeley, Berkeley, California, United States of America; University of Groningen, NETHERLANDS

## Abstract

Individuals prone to anxiety and depression often report beliefs and make judgements about themselves that are more negative than those reported by others. We use computational modeling of a richly naturalistic task to disentangle the role of negative priors versus negatively biased belief updating and to investigate their association with different dimensions of Internalizing psychopathology. Undergraduate participants first provided profiles for a hypothetical tech internship. They then viewed pairs of other profiles and selected the individual they would prefer to work alongside out of each pair. In a subsequent phase of the experiment, participants made judgments about their relative popularity as hypothetical internship partners both before any feedback and after each of 20 items of feedback revealing whether or not they had been selected as the preferred teammate from a given pairing. Scores on latent factors of general negative affect, anxiety-specific affect and depression-specific affect were estimated using participants’ self-report scores on standardized measures of anxiety and depression together with factor loadings from a bifactor analysis conducted previously. Higher scores on the depression-specific factor were linked to more negative prior beliefs but were not associated with differences in belief updating. In contrast, higher scores on the anxiety-specific factor were associated with a negative bias in belief updating but no difference in prior beliefs. These findings indicate that, to at least some extent, distinct processes may impact the formation of belief priors and in-the-moment belief updating and that these processes may be differentially disrupted in depression and anxiety. Future directions for enquiry include examination of the possibility that prior beliefs biases in depression might reflect generalization from prior experiences or global schema whereas belief updating biases in anxiety might be more situationally specific.

## Author summary

Pessimistic beliefs are at the core of both depression and anxiety. How do these beliefs form and is this process similar or different for depression and anxiety? Here, we investigate these questions in a naturalistic experimental setting, in which undergraduate participants competed against one another to be selected by their peers in a hypothetical tech internship and were asked to judge their popularity relative to other participants. Importantly, participants were asked to make these judgements before any feedback and after receiving a series of pieces of feedback about whether or not they had been selected over other specific students. This design, along with computational modelling of the formation of participants’ beliefs, allowed us to establish that anxiety is associated with pessimistic beliefs that arise through negatively biased belief updating–i.e., the incorporation of negative information more readily into one’s beliefs than positive information–while depression is associated with pessimistic prior beliefs–that is, beliefs that are formed before receiving explicit feedback and likely on the basis of prior experiences or pre-existing schema.

## Introduction

When asked to judge the probability that they will experience a range of future positive events, individuals suffering from depression give lower probability estimates than non-depressed individuals [1,2]. Both depression and anxiety have also been associated with elevated self-referential probability judgements for negative events [3,4]. Why might this be the case? Cognitive theories of depression have suggested that global negative self-referential beliefs might arise as a result of dysfunctional schema developed during childhood with these beliefs being generalized across, and applied to, many different situations [5–7]. In computational terms, these beliefs may be thought of as priors. Individuals may also differ in the extent to which they accommodate new information that is either positively or negatively valenced [8,9]. This would constitute asymmetric learning. Here, we designed a task that would allow us to distinguish the relative contribution of priors and asymmetric learning to biased beliefs in depression and anxiety.

Much of the work investigating biases in belief updating has been conducted using judgements of the probability of experiencing various potential life events [10–13]. In these studies, individuals are asked to estimate the probability that they would experience a future event (e.g., being diagnosed with cancer), they are then given information about the population base rate, and finally they are asked to revise their beliefs. Healthy individuals tend to show positively biased updating (i.e., towards ‘good news’ rather than ‘bad news’ [14]), while depressed patients show an absence of this positivity bias [11,13]. This approach has attracted criticism [15] on statistical grounds, as, for instance, the experimenter may not have access to information that might influence the relevance of a given base rate to a given participant’s belief (e.g., a family history of cancer might lead an individual to estimate their own risk as higher than the population-wide base-rate) and such information might impact extent of belief updating.

Recognition of the need for greater experimental control has led to alternative experimental paradigms being created to assess belief updating. One approach has been to use novel, emotionally-neutral stimuli in an associative-learning paradigm and to examine updating of explicit expectations associated with these stimuli. For example, in [16], healthy individuals were asked to update their beliefs about the probability of reward associated with arbitrary shapes in response to reward feedback. Trait optimism was not found to be associated with biased updating. The authors posited that this null result might reflect the stimuli being highly removed from personal concerns. Indeed, clinical studies have reported that cognitive biases are more likely to be observed when scenarios or stimuli align with individuals’ concerns [17].

An alternate approach to examining belief-updating seeks to achieve a balance between experimental control and ecological validity. Here, studies conducted by Eil & Rao (2010) and Mobius et al. (2010) provide initial exemplars upon which we build [18,19]. Eil & Rao (2010) asked healthy adult participants to estimate their rank relative to other participants for performance on an IQ test and how highly other participants rated them in terms of physical attractiveness [18]. Participants were told whether or not they ranked higher or lower than another randomly selected participant and were then asked about their updated beliefs. Participants incorporated negative feedback into their beliefs to a lesser extent and less reliably than positive feedback, demonstrating a positive bias. Mobius et al. (2010) gave participants probabilistic feedback about whether they were in the top half of performers on an IQ test and observed that participants asymmetrically updated more for positive than for negative information [19].

In the current study, we similarly chose an experimental design aimed at providing both experimental control and ecological validity. We ensured that the accuracy of initial beliefs could be verified as these pertained to judgements made by other participants in an earlier stage of the experiment. We also ensured that structurally equivalent other-referent judgements could be obtained. In addition, the information provided to participants after assessment of their initial belief was unique to them (or the specified ‘other’) and to the current scenario and hence less likely to be discounted as an irrelevant base rate. We built a computation model of the data collected to estimate both initial beliefs and to investigate asymmetry in belief updating. Finally, we used bifactor modeling of anxiety and depressive symptoms to determine whether biases in initial beliefs or in belief updating were common to both anxiety and depression, unique to depression or unique to anxiety.

## Results

### Overview

In the San Francisco Bay Area, university students frequently compete for prestigious tech internships. They are also familiar with working in small teams and being judged on the work produced. We therefore designed our experiment to investigate participants’ beliefs about their desirability as a potential team partner for an internship project. All participants were UC Berkeley undergraduates. The experiment comprised three sessions. In the first, we asked participants to provide us with internship profiles, and in the second, we asked participants to select preferred teammates out of a series of pairs of profiles provided by other students. In the final part, we assessed students’ prior beliefs as to their popularity as a potential team partner and examined how these beliefs were updated as a function of sequential feedback. In each of the three sessions, participants completed standardized questionnaire measures assessing anxiety and depressive symptoms.

### Characterizing participants in terms of scores on latent dimensions of anxiety and depressive specific affect and general negative affect

Participants completed four questionnaires that covered a wide range of depression and anxiety symptomatology. These comprised the Penn-State Worry Questionnaire (PSWQ; [20]), the Mood and Anxiety Symptom Questionnaire (MASQ; [21,22]), the Spielberger State-Trait Anxiety Inventory (STAI form Y; [23]) and the Center for Epidemiologic Studies Depression Scale (CESD; [24]); see Methods for more details. We report the mean and standard deviation of participants’ scores on these questionnaires in S1 Table and compare these scores to those from individuals diagnosed with Generalized Anxiety Disorder (GAD) or Major Depressive Disorder (MDD), age and gender matched controls, and a community sample recruited for a prior study [25].

In our previous work [25], we have conducted a bifactor analysis on item-level responses to these and other measures of anxiety and depression to separate symptom variance common to both anxiety and depression from that specific to depression or to anxiety. The specific form of this bifactor model was informed by tripartite theories of anxiety and depression (e.g., [21]) and consists of three orthogonal factors: a general factor and two specific factors. The general (‘negative affect’) factor captures symptom variance shared between anxiety and depression. The two specific factors capture symptom variance specific to anxiety and to depression, respectively. In the current study, we used the factor loadings for items belonging to the PSWQ, CESD, STAI-trait scale and MASQ anhedonic and anxious arousal subscales to calculate scores on the anxiety- and depression-specific factors and the general negative affect factor for each of our current participants. We re-analyzed the data published in Gagne et al (2020) [25] to establish the equivalence of factor scores obtained using this subset of items to those derived using the full original set (see Methods for details). S1 Fig presents the correlations between the resultant factor scores for our current participants and summary scores for the questionnaire subscales completed. As anticipated, scores on the depression-specific factor were strongly correlated with scores on subscales tapping anhedonic symptoms of depression (MASQ anhedonic depression: r(64) = 0.74; CESD anhedonic depression session 2: r(64) = 0.66; CESD anhedonic depression session 3: r(64) = 0.49). There was also a selectively strong correlation between scores on the anxiety-specific factor and the PSWQ (r(64) = 0.78). In our prior work, we also found that scores on the anxiety-specific factor loaded more strongly onto the PSWQ than any other measure of anxiety [25]. The general negative affect factor showed strong correlations across many subscales tapping both anxious and depressive symptomatology (highest loadings: CESD somatic symptoms session 3, r(64) = 0.77; STAI anxiety session 3, r(64) = 0.75; STAI anxiety session 2, r(64) = 0.71; MASQ general depression symptoms, r(64) = 0.66; CESD somatic symptoms session 2, r(64) = 0.64; MASQ general anxiety symptoms, r(64) = 0.63).

### Data acquired to assess prior beliefs and belief updating

**Fig 1:**
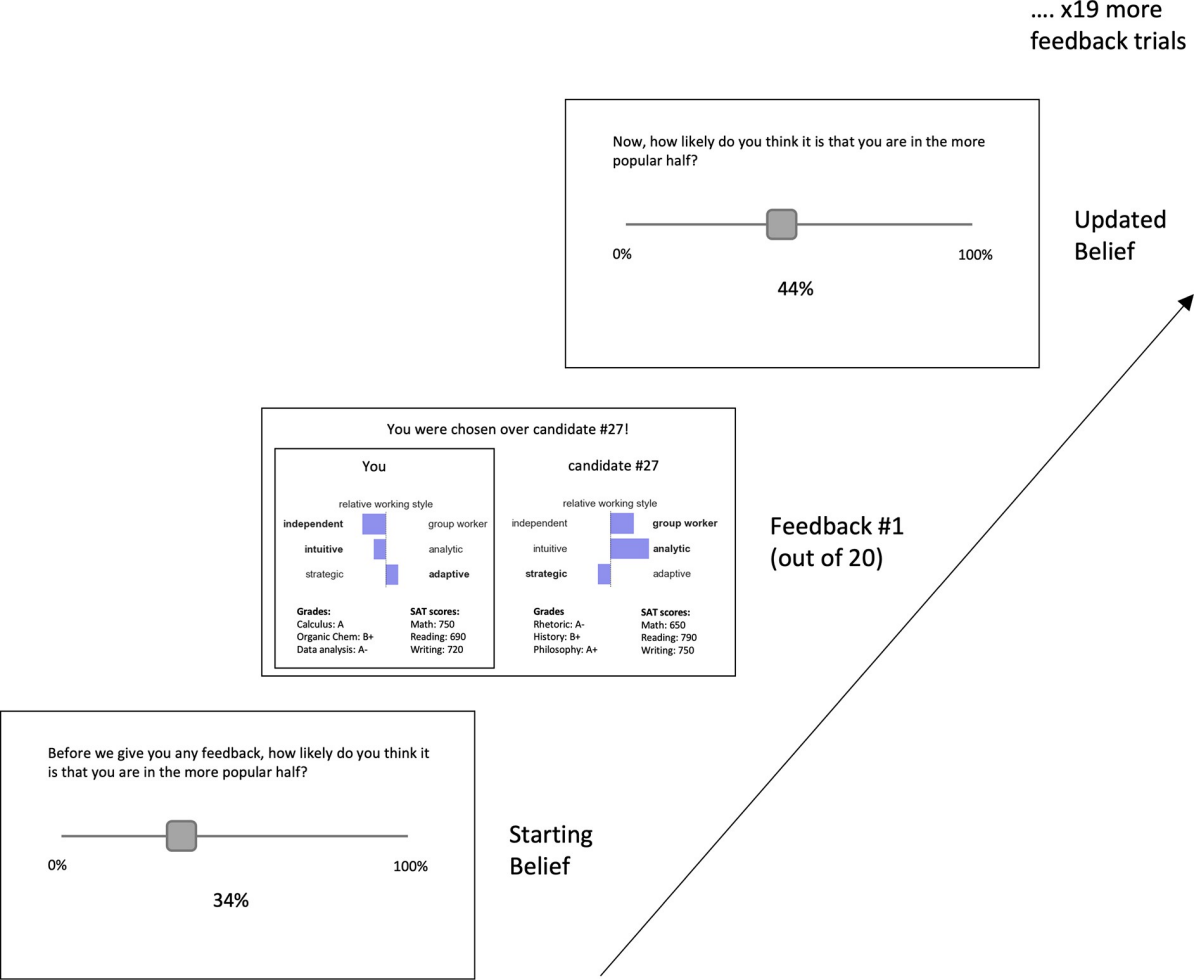
Experimental Session 3. Before receiving any feedback from session 2, participants were asked to use a slider (bottom left) to estimate the probability that they were in the more popular half of students. Participants were then shown a pair of profiles, containing their profile and another participant’s profile, and whether or not they had been chosen by a third participant during session 2 (depicted by a black outline, see central display). After receiving this piece of feedback, participants were again asked to estimate the probability that they were in the more popular half of students (top right). Participants were given twenty pieces of feedback in total and were asked to report their updated judgement following each piece. Participants then repeated this task but observing an anonymous ‘other’ participant whose profile they were shown.

The task consisted of three parts. In part one, each participant made a ‘profile’, consisting of their actual grades, SAT scores, and a brief description of why they would be a good teammate during internship (see Methods). In part two, participants were shown pairs of other participants’ profiles and were asked to choose with which of the pair they would rather work. Every participant’s profile was paired with at least 30 other profiles. At the start of part three, we assessed participants’ belief as to the probability that they were in the top (or bottom) half of participants in terms of how often they had been chosen as a potential team partner. We then presented 20 sequential pieces of feedback, each indicating whether they were chosen versus another (anonymized) candidate (see Fig 1). No pairing was repeated. After each piece of feedback, participants were again asked to judge the probability that they were in the top (or bottom) half of participants. These twenty pairs were selected from all the possible pairs in which the participants’ profile had been included to achieve a balanced set of ten instances of positive feedback and ten instances of negative feedback for each participant. Positive feedback indicated that they were chosen over another participant as a potential internship partner; negative feedback indicated that another participant was chosen over them. This feedback was delivered using one of two pre-randomized sequences (see Methods).

After assessing their own probability of being in the top 50% of potential internship partners, participants were asked to perform an equivalent judgement task for an unnamed ‘other’ participant whose profile they were shown. For each participant, we chose a profile of similar ‘competitiveness’ (i.e., ranking in terms of how often they were chosen) to the participant. Participants were first shown the anonymized profile and asked to estimate the probability that the participant was in the more popular half of students. They were then given ten instances of positive feedback and ten instances of negative feedback (i.e., profile pairings where the other individual had or had not been selected) using the same feedback order sequence as allocated to them for the self-referential version of the task, see Methods.

### Model construction and comparison

We modeled the data collected in part 3 to estimate participants’ prior beliefs and the extent to which these were updated as a result of feedback. Here, we describe model construction and comparison and present the results from the winning model. Parallel model-agnostic analyses were also conducted. We briefly detail whether these do, or do not, replicate the findings using the more sensitive model fitting approach but leave full presentation of these analyses to S1 Text.

The models were designed to estimate prior beliefs, trial to trial changes in belief updating, and the presence or absence of biases, and to allow us to characterize the relationship of each of these components with anxiety, depression, and general negative affect. The models share a common architecture for the way that beliefs are represented and reported; they differ in their specific mechanisms for belief updating. Since the belief of interest is a probability over a binary outcome (that subjects were in the top half of participants), we adopted a conventional representation of beliefs as a Beta distribution *B*(*α*_*t*_, *β*_*t*_) on trial *t*≥0. This offers a natural way to parameterize subjects’ initial and evolving uncertainty about their beliefs, with the two Beta distribution parameters *α*_*t*_, *β*_*t*_ acting as subjective estimates of the weight of evidence in favour of, or against, the probability of being in the top-half of the participants, respectively. We considered subjects’ reports, ${\hat{q}}_{t}∼B(\alpha_{t},\beta_{t})$, at any point *t* to be a single sample from their Beta distribution at that point. This approach offers a simple account of variability in subjects’ reported beliefs without requiring any additional parameters; for prior examples of this approach see [26–28].

Based on this common architecture, we tested four alternate specific models that make different assumptions as to how participants update their Beta distribution in response to the feedback they receive over the course of the 20 trials in part 3. These four models varied as to (i) whether the parameters of the distribution were updated according to Bayesian or Rescorla-Wagner (RW) principles and (ii) whether a bias in updating after negative versus positive feedback was incorporated (see Methods for full details of each model). We used exceedance probabilities to compare model fit [29]. The winning model was the one in which distribution parameters were updated according to RW principles and which allowed for asymmetric updating after negative versus positive feedback (Model 3; exceedance probability > 0.99). In this model, the Beta distribution is determined according to its mean (*μ*_*t*_) and a constant precision (*ν*; i.e., belief certainty). The mean starts at *μ*_0_ and is then updated directly in response to feedback (*X*_*t*_). Positive, relative to negative, updating is scaled by a bias term (*b*), with *b*>1 favoring evidence of being selected and *b*<1, evidence of being rejected, and occurs using a learning rate (*η*), with the constraint that *μ*_*t*_ is not allowed to exceed 1 (see Eq 1).


$$\mu_{t}=min(\mu_{t-1}+\eta ({bX}_{t}-\mu_{t-1}),1)$$ (1)

The parameters of the Beta distribution are then derived as *α*_*t*_ = *νμ*_*t*_; *β*_*t*_ = *ν*(1−*μ*_*t*_), and the report on trial *t*≥0 is given by a single sample ${\hat{q}}_{t}∼B(\alpha_{t},\beta_{t})$. This model has four free parameters: *μ*_0_∈[0,1], *ν*∈[2,1000], *η*∈[0,1], *b*∈[0,5].

Simulating data further revealed that the biased RW model best reproduced the distribution of participants’ average belief updates following positive and negative feedback (see S2 Fig).

We also compared the winning model against a number of additional alternate models (see S2 Text). Among this larger set of models, Model 3 still had the highest exceedance probability (exceedance probability = 0.67, S3 Fig). The results of a model identification analysis revealed that model 3 was distinguishable from all other models tested (S4 Fig).

### Biases in prior beliefs: group level analyses

The winning model (Model 3: biased RW) estimates participants’ initial, or prior, belief about whether they were in the top half of participants, in terms of popularity as a hypothetical internship partner, using the mean (*μ*_0_) of the initial belief distribution; i.e., the mean at time 0 prior to the first instance of feedback. In previous research, the finding that more than half of respondents rate themselves as being in the top half for a given skill (e.g., driving ability; [30]) has been taken as evidence of a prevalent optimism bias (reviewed in [10]). In the current study, participants’ initial belief, as estimated by *μ*_0_, did not reveal evidence for any systematic bias at the group level; mean probability of being in top half = 0.498, t(64) = -0.065, p = 0.948. There was also no effect of framing—that is, whether participants were asked to judge whether they were in the top or bottom half—on initial beliefs *μ*_0_, t(64) = -0.012, p = 0.99. We note that model agnostic analyses confirmed these findings (see S1 Text).

### Biases in prior beliefs: relationship with Internalizing symptoms

**Fig 2:**
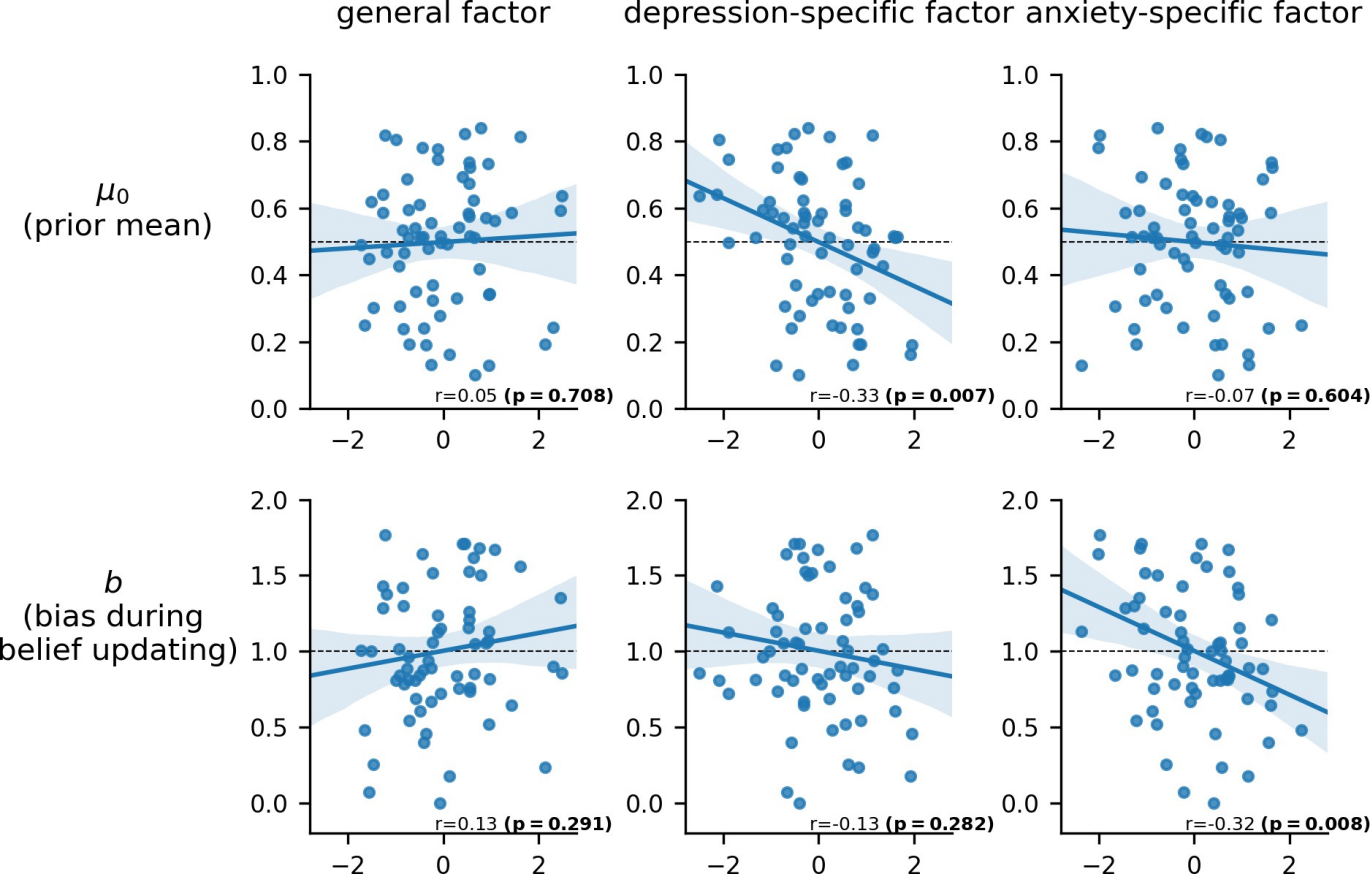
Relationship between parameter estimates for the prior mean (*μ*_0_) and bias in belief updating (*b*) and depression-specific and anxiety-specific factor scores. Participants’ standardized scores for the general factor, anxiety-specific factor and depression-specific factor (x axes) are plotted against parameter estimates for the prior mean (*μ*_0_) and bias in belief updating (*b*), obtained using the winning model: Model 3 the “biased RW” model. *μ*_0_ is the estimated mean of the participant’s belief distribution at the start of the feedback period and *b* represents bias in updating in response to positive or negative feedback (*b*>1 corresponds to a positive bias, whereas *b*<1 corresponds to a negative bias). P-values for Pearson correlations were obtained using two-tailed permutation-tests with 10,000 samples.

We next turned to the question of whether high levels of Internalizing symptoms were associated with more negative prior beliefs. We examined this for each of our three latent dimensions of Internalizing symptoms: general negative affect, depression-specific affect and anxiety-specific affect. Here we report the results of Pearson correlations; two-tailed permutation-tests with 10,000 samples were used to calculate the significance of each correlation reported (see Methods). Participants’ prior belief that they were in the most popular half of participants, as modeled by *μ*_0_ scores, significantly negatively correlated with their score on the depression-specific factor, r(64) = -0.33, uncorrected p = 0.007, Fig 2. This relationship survived when controlling for the total number of tests needed to examine the relationship between each of the four model parameters and each of the three latent measures of negative affect (i.e. twelve tests, FDR pcorr = 0.048). There was no significant relationship between *μ*_0_ and either anxiety-specific or general negative affect factor scores, r(64) = -0.07, uncorrected p = 0.60, FDR corrected p = 0.72; r(64) = 0.05, uncorrected p = 0.71, FDR corrected p = 0.77. A supplementary analysis confirmed that there was no significant relationship between Internalizing symptom scores and the effect of framing on starting beliefs |β|<0.025, ps>.65, uncorrected. Model agnostic analyses produced consistent findings; elevated scores on the depression-specific factor, alone, were significantly linked to negatively biased starting beliefs (see S2 Table and S1 Text). These analyses also confirmed the absence of any interaction with framing.

### Biases in belief updating: group level analyses

The degree to which participants update their beliefs asymmetrically, whether more strongly after positive or negative feedback, is estimated in Model 3 by the bias parameter *b*. Across participants, there was no evidence of positively biased (*b*>1) or negatively biased (*b*<1) updating; the mean value for *b* did not significantly differ from 1 (mean = 1.003; t = 0.05, p = 0.95). However, there was a wide range of individual differences in the estimated bias parameters (std. deviation of 0.44), indicating that our participant sample included both participants who updated more strongly after positive feedback and participants who updated more strongly after negative feedback.

### Biases in belief updating: relationship with Internalizing symptoms

Across participants, the updating bias parameter, *b*, was significantly correlated with anxiety-specific factor scores, r(64) = -0.32, uncorrected p = 0.008, FDR corrected p = 0.048. This indicates that high levels of anxiety-specific affect are associated with negatively biased updating. In contrast, neither depression-specific or general negative affect factor scores showed a significant relationship with the bias parameter, r(64) = -0.13, uncorrected p = 0.28, FDR corrected p = 0.50; r(64) = 0.13, uncorrected p = 0.29, FDR corrected p = 0.50, respectively. In the model-agnostic analysis, we used the extent to which participants changed their probability estimates from before feedback to after all feedback to index belief updating. Here, we observed only a trend-level relationship (p = 0.065 uncorrected) between anxiety-specific factor scores and the extent to which belief updating was negatively biased, S2 Table. That this model-agnostic analysis did not reveal the significant relationship between anxiety and asymmetric updating captured by the RW-updating-with-bias computational model potentially speaks to the increased sensitivity available from modeling trial-to-trial changes in beliefs.

### Other model parameters

The learning rate parameter *η* in Model 3 estimates the absolute amount that participants update their beliefs following feedback (independently of the feedback’s valence). The mean learning rate was 0.098, which means that on average participants revise their current belief by approximately 10% per trial using the feedback. Participants’ reported beliefs were estimated to be narrowly distributed around their mean, with an average precision parameter of *ν* = 320, across participants. For a neutral belief of 0.5, this precision value would correspond to a *B* (*α*_*t*_ = 160, *β*_*t*_ = 160) distribution, which has a standard deviation of only 0.03. Across participants, neither learning rate nor precision values significantly correlated with scores on any of the three Internalizing symptom factors (see S5 Fig).

### Confirmation biases: exploring the relationship between prior beliefs and updating asymmetry

**Fig 3:**
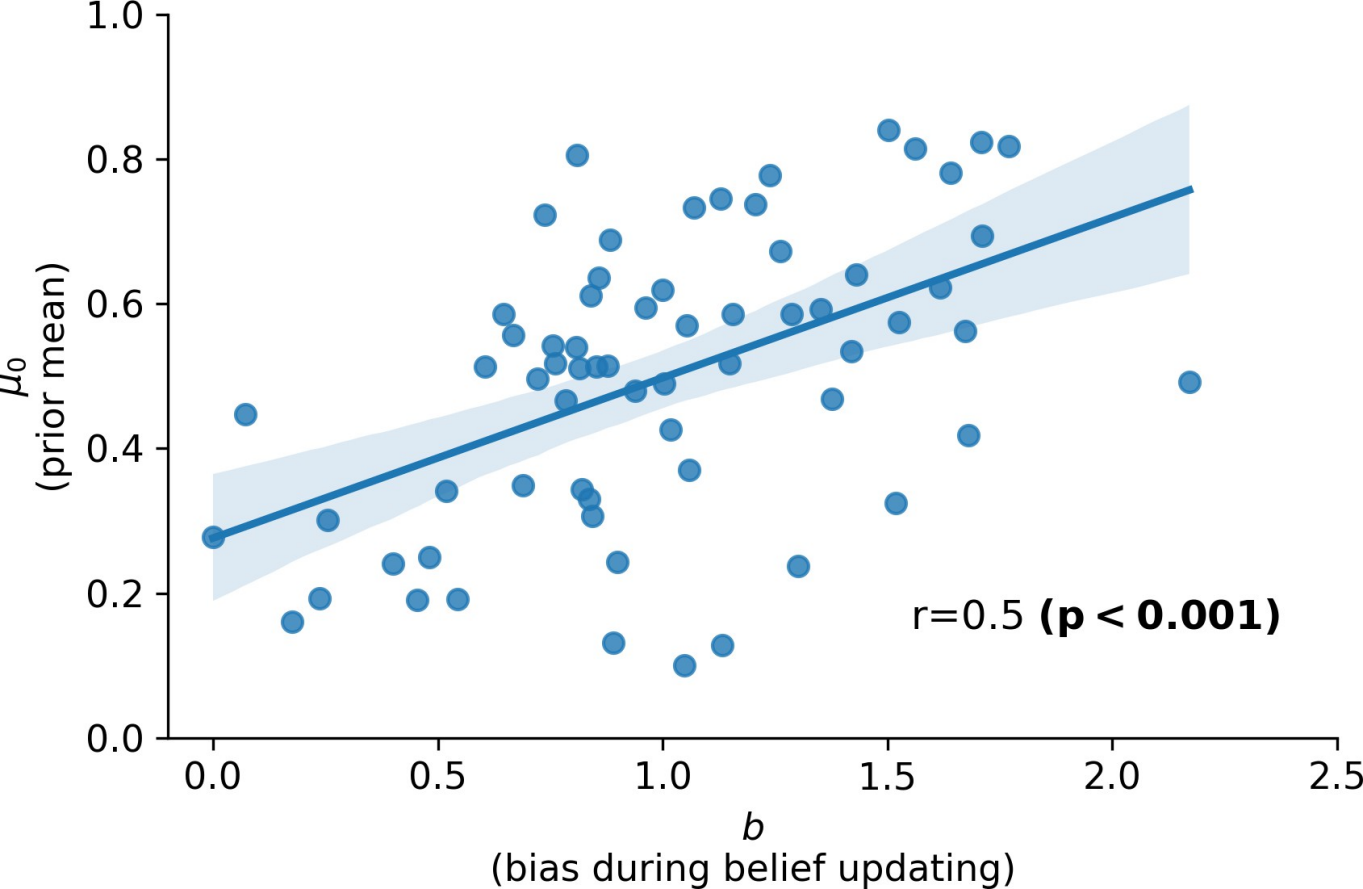
Evidence for a confirmation bias for self-referential beliefs. Parameter estimates for the prior mean (*μ*_0_; y-axis) and bias in belief updating (*b*; x-axis), obtained using the winning model (Model 3: the “biased RW” model), are significantly correlated across participants (r = 0.5, p<0.001). This can be thought of as a form of confirmation bias, with new information (positive versus negative feedback) being incorporated more strongly into participants’ beliefs when it aligns with prior beliefs.

In this experiment, the combination of a negative prior belief (*μ*_0_<0.5) and negatively biased updating (*b*<1) or a positive prior belief (*μ*_0_>0.5) and positively biased updating (*b*>1) would give rise to a *confirmation bias*–that is new information is incorporated selectively in a manner that matches a priori biases. As shown in Fig 3, across participants, estimates for *μ*_0_, that is participants’ prior belief that they were in the most popular half of participants, showed a strong positive correlation with the updating bias *b* parameter.

Given this significant relationship between the prior belief (*μ*_0_) parameter and updating bias (*b*) parameter across participants, a key question is whether we observe the relationship between depression and prior belief (*μ*_0_) parameter estimates and between anxiety and updating bias (*b*) parameter estimates when the relationship between latent factor scores and both parameters are considered simultaneously. To address this, we conducted three additional regression analyses with scores on the latent Internalizing factors as dependent variables and parameter estimates for both *μ*_0_ and *b* as predictors. These additional regressions (see S3 Table) confirmed our original findings of a significant relationship between *μ*_0_ and depression-specific affect and between *b* and anxiety-specific affect. Direct comparison of coefficient values indicated that *μ*_0_ was a significantly stronger predictor of scores on the depression-specific factor than of scores on the anxiety-specific factor (difference in beta-coefficient for *μ*_0_ between depression and anxiety regression models = -2.45; p = 0.0059 by permutation test with 10,000 samples), and that *b* was a significantly stronger predictor of scores on the anxiety-specific factor than of scores on the depression-specific factor (difference in beta-coefficient for *b* between anxiety and depression regression models = -0.96; p = 0.0139 by permutation test). Neither *μ*_0_ nor *b* significantly predicted scores on the general negative affect factor, p>.3. Adding the interaction term for *μ*_0_ by *b* did not significantly improve the fit of the regression models though there was a non-significant trend in this direction for depression-specific scores, likelihood ratio test, p = 0.064. Here, there was a non-significant trend for the relationship between negative prior beliefs and elevated depression scores to be amplified in individuals with a negative updating bias, p = 0.074.

### Prior Beliefs and Ground Truth: Depressive Realism?

An important question is whether the negative starting beliefs held by participants high in depression reflect negatively biased priors or accurate self judgements. A number of studies have suggested that the negative beliefs held by individuals with depression may be more accurate than the positive, potentially ‘optimistically’ biased, beliefs held by healthy individuals [31]. Furthermore, if individuals with high depression-specific factor scores are chosen less often as teammates, their negative beliefs might be accurate even if their judgements are not higher in accuracy than those of individuals low in depression levels.

**Fig 4:**
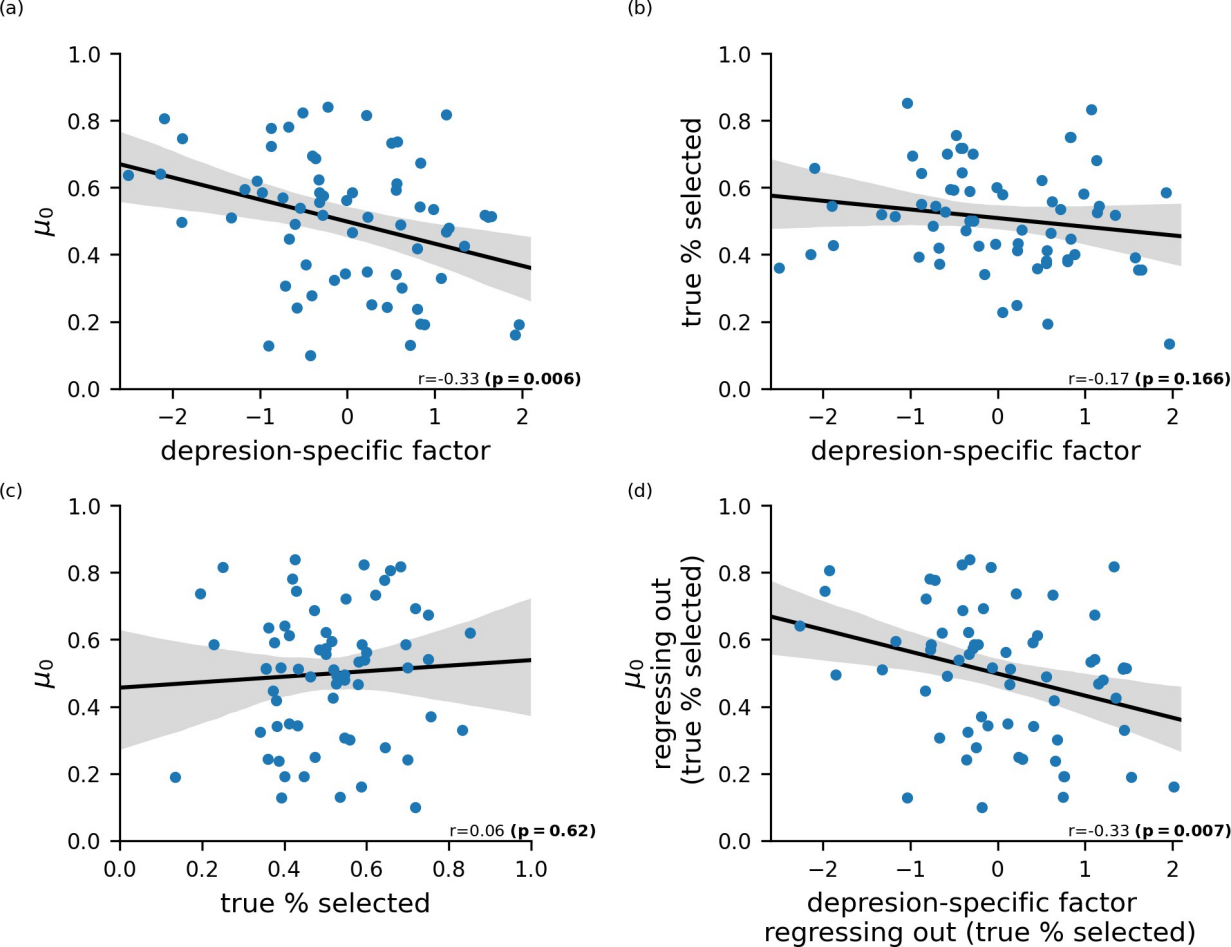
Testing for Depressive Realism. We used the percentage of times that a participant was actually chosen as a potential partner in session 2 (‘true % selected’) to investigate if depression-related differences in starting beliefs (*μ*_0_) might actually reflect accurate perceptions of genuine differences in ‘profile popularity’, i.e., popularity as a potential internship partner. (a) Scores on the depression-specific latent factor were significantly negatively correlated with starting beliefs, r(64) = -0.33, p = 0.006. (b) Scores on the depression-specific factor showed a non-significant negative association with profile popularity (as indexed by true % selected, y-axis), r(64) = -0.17, p = 0.17. (c) Participants had poor insight into their profile popularity; correlation between true % selected and starting belief: r(64) = 0.06, p = 0.62. (d) Partialing out profile popularity (i.e., the true % selected for each participant) had little impact on the significant relationship between depression-specific factor scores and starting beliefs (partial r = -0.33, p = 0.007). A causal mediation analysis [32] confirmed that the relationship between scores on the depression-specific factor and negative starting beliefs was not mediated by profile popularity; Average Causal Mediation Effect (ACME) = 0.0002, p = 0.97.

An advantage of our experimental design is that we can examine the accuracy of participants’ starting beliefs by measuring the percentage of times that a participant was actually chosen as a potential partner in session 2. This gives us an index of ‘profile popularity’. Profile popularity did not mediate the relationship between scores on the depression-specific factor and starting beliefs *μ*_0_ (p = 0.97; causal mediation analysis, [32]; see Fig 4). If we examine this in more details, we find that scores on the depression-specific factor were not significantly negatively correlated with profile popularity, r(64) = -0.17, p = 0.17. Further, there was not a significant correlation between profile popularity and starting belief (*μ*_0_) across participants (r(64) = 0.06, p = 0.62) indicating that participants did not possess good insight into how popular they would be as hypothetical internship partners. There was also no significant relationship between depression-specific factor scores and accuracy at predicting one’s own profile popularity, r(64) = -0.02 p = 0.86.

Turning to our other measures of latent affect; anxiety was not associated with profile popularity, r(64) = 0.04, p = 0.72. Higher scores on the measure of general negative affect showed a trend-level negative relationship with profile popularity, r(64) = -0.21, p = 0.084. Neither anxiety nor general negative affect were significantly associated with accuracy at predicting one’s own profile popularity, r(64) = 0.16, p = 0.21; r(64) = 0.16, p = 0.21, respectively.

### A model-based analysis of other-referent beliefs

After assessing their own probability of being in the top 50% of potential internship partners, participants were asked to perform an equivalent judgement task for an unnamed ‘other’ participant whose profile they were shown. For each participant, we chose a profile of similar ‘competitiveness’ (i.e., ranking in terms of how often chosen) to the participant. Participants were first shown the anonymized profile and asked to estimate the probability that the participant was in the more popular half of students. They were then given ten instances of positive feedback and ten instances of negative feedback (i.e., profile pairings where the other individual had or had not been selected) using the same feedback order sequence as allocated to them for the self -referent version of the task.

### Other-referent beliefs: model comparison results

We considered the same four models used to fit participants self-referential belief data and applied them to each participants’ other-referent belief data. The best fitting model for other-referent beliefs was again Model 3 (exceedance probability > 0.99; when comparing the four primary models). When a larger set of models was considered, in parallel to our supplementary model comparison procedures for participants’ self-referential belief data, Model 3, once again, remained the most likely model with an exceedance probability of 0.62; **S6 Fig**). We note that the models with the second and third highest exceedance probability differed between the self-referential belief data set and the other-referential belief data set. In the supplements we report the results of fitting the second and third-best models and show that the anxiety and depression results reported for both self and other beliefs are robust to the model chosen, see S2 Text.

### Initial other-referent beliefs: group level analyses

Intriguingly, participants showed a positive bias in their prior belief (*μ*_0_) for other-referent judgements; mean probability that other in the top half = 0.55, t-test against neutral belief (0.5), t(65) = 3.09, p = 0.002. A paired t-test for self-referent versus other-referent judgements confirmed that, at a group-level, participants were more likely to estimate that the selected unknown ‘other’ participant was within the top half of participants than they were for themselves, t(65) = -2.02, p = 0.047. This is despite the ‘other’ profile used having been selected uniquely for each participant to be well matched to them in popularity (paired t-test, self versus other popularity; t(65) = -1.38, p = 0.17; note that despite popularity being slightly lower for self than for other, the self-other difference in initial beliefs survives controlling for popularity; mixed-effects model predicting initial beliefs; self-other *β* = -0.055, p = 0.041). As was the case for self-referential judgements, there was no influence of framing (probability in top or bottom half) on initial beliefs, p>0.4. This finding reveals that our participants, as a group, showed a negative or ‘pessimistic’ bias in initial assessments of their own popularity relative to an unknown participant of similar social standing.

### Initial other-referent beliefs: relationship with Internalizing symptoms

Scores on the depression-specific factor were not significantly correlated with participants’ initial other-referent belief, though there was a trend towards a negative correlation (r(64) = -0.23; uncorrected p = 0.066). The relationship between depression-specific factor scores and the difference in initial beliefs for self versus other did not reach significance, r(64) = -0.17, uncorrected p = 0.17.

### Other-referential belief updating

At a group level, participants showed a positive bias when updating their other-referent beliefs after feedback, bias parameter *b* mean = 1.16, t(64) = 2.7, p = 0.008. That participants had a more positive updating bias when making judgments for the selected unknown ‘other’ participant than for themselves was confirmed with a paired t-test, t(64) = -2.43, p = 0.018. This suggests, along with positively biased initial estimates, that participants tend to show more of an ‘optimism bias’ for others than for themselves. There was no effect of feedback order on the updating bias parameter *b*, for other-referential beliefs, t(64) = 1.6, p = 0.10.

### Other-referential belief updating: relationship with Internalizing symptoms

In contrast to self-referential belief updating, scores on the anxiety-specific dimension of affect did not show a significant relationship with the updating bias parameter for other-referential beliefs, r(64) = -0.06, p = 0.61. However, the correlation between scores on the anxiety-specific factor and the difference in bias parameter scores (*b*) for self- versus other- referential belief updating did not reach significance, r(64) = -0.21, uncorrected p = 0.10. In addition, no significant relationship was observed between *b* and the general negative affect or depression-specific affect scores (r(64) = -0.05, p = 0.71; r(64) = -0.04, p = 0.72), or between these factors and the difference in bias parameters for self- versus other-referential beliefs, r(64) = 0.14, p = 0.23; r(64) = -0.07, p = 0.58.

### Other-referential beliefs: confirmation biases

**Fig 5:**
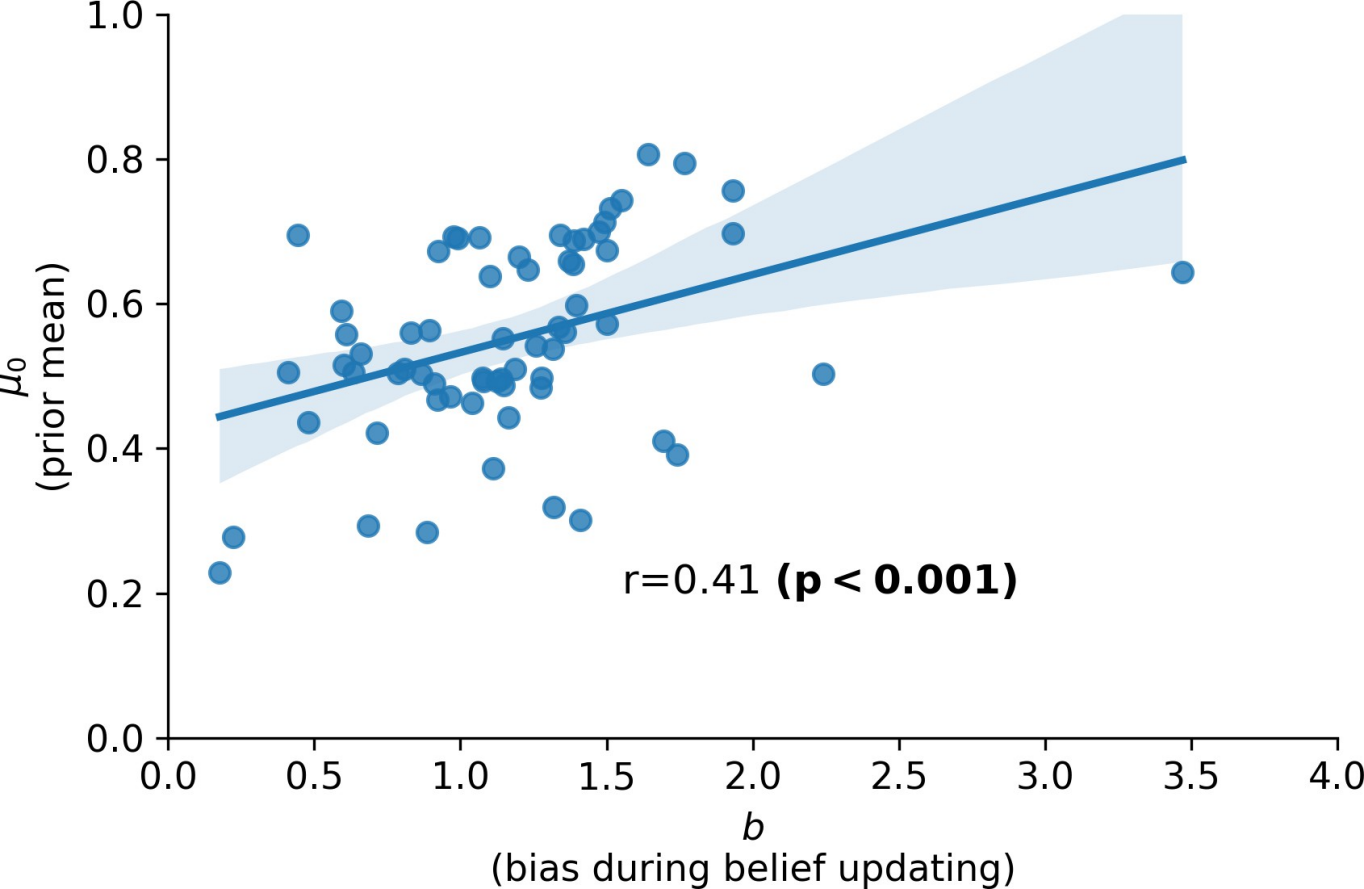
Evidence for a confirmation bias for other-referent beliefs. Parameter estimates for the prior mean (*μ*_0_; y-axis) and bias in belief updating (*b*; x-axis), obtained using the winning model (Model 3: the “biased RW” model), are significantly correlated across participants (r = 0.41, p<0.001); this relationship also holds without the outlier, r = 0.44, p<0.001). Thus, participants, as a group, tend not only to show a confirmation bias when updating beliefs related to themselves (as shown in Fig 4) but also when updating their beliefs about other participants. Here, a confirmation bias means that positive/negative feedback is incorporated more strongly into beliefs when it aligns with prior beliefs.

Interestingly, we also saw evidence for confirmation biases in participants’ other-referential belief data, as evidence by a positive correlation between prior beliefs (positive versus negative) and updating bias (positive versus negative), Fig 5.

Given this significant relationship between the prior belief (*μ*_0_) parameter and updating bias (*b*) parameter across participants, we conducted additional regression analyses with scores on the three latent factor dimensions as dependent variables and parameter estimates for both *μ*_0_ and *b* as predictors. These additional regressions (see S4 Table) confirmed the findings from our correlational analyses, with neither *μ*_0_ or *b* for other-beliefs significantly predicting scores on any of the three latent dimensions, with only a trend-level relationship between *μ*_0_ parameter estimates and depression-specific affect, (t = -1.870, p = 0.066).

Including the interaction term for *μ*_0_ by *b* in prediction of latent factor scores did not significantly improve regression model fit (relative to the model with terms for *μ*_0_ and *b* alone) and revealed no significant effect of *μ*_0_×*b* for anxiety, depression or general negative affect factor scores.

## Discussion

In this study, we used a peer selection task to examine two potential sources of negative self-referential judgements in undergraduates with high levels of anxiety and depression: negative priors and negatively biased belief updating. Given the extent of shared variance between scores on self-report measures of anxiety and depression, we used a bifactor analysis of item-level responses [25] to estimate participants’ scores on three orthogonal latent factors: an anxiety-specific factor, a depression-specific factor, and a general negative affect factor. Participants’ judgements as to the probability that they were among the top or bottom half of participants in terms of how frequently they were chosen as a potential internship partner were assessed before feedback and after each of 20 items of feedback. These judgements were modeled as samples from an underlying belief distribution, using Rescorla-Wagner based updating and a bias parameter to capture asymmetric updating. Scores on the latent factor of depression-specific affect were significantly correlated with the negativity of participants’ prior beliefs as to their own popularity. There was no significant relationship between depression-specific affect and the updating bias parameter. In contrast, elevated scores on the latent factor of anxiety-specific affect were linked to biased belief updating, with a significantly higher rate of updating following negative than positive feedback. Here, there was no significant relationship with prior beliefs. Scores on the general negative affect dimension were not significantly associated with either negativity of prior beliefs or biased updating of beliefs.

At a group-level, we observed a strong relationship between prior beliefs and updating biases–that is participants who showed more negative prior beliefs also tended to update their beliefs more strongly after negative feedback and participants who showed more positive prior beliefs tended to update their beliefs more strongly after positive feedback. Regression analyses confirmed that the relationship between elevated depression-specific affect and prior negativity and between anxiety-specific affect and negatively biased updating remained significant when both prior belief and updating bias parameter estimates were included as predictors of latent factor scores. A way to think about this is that, at a group level, these influences of anxiety and depression are effectively super-imposed on a normative tendency for individuals to show consistency between their initial beliefs and updating style.

We also asked participants to perform a parallel rating task for another anonymized participant of equivalent objective popularity. None of the three latent factors was significantly associated with positive or negative biases in prior beliefs or belief updating for these other-referential judgements. As a group, participants showed a positivity bias in both prior beliefs and belief updating for other-referential judgements that was absent in relation to their self-referent judgements.

In the clinical literature of the 1980’s and 1990’s, there was substantial research as to the relationship between negative affect and judgements of the probability of future events. These studies revealed that both anxiety and depression were associated with elevated judgements of the future probability that the participant would, themselves, experience a range of negative events [3]. This was not observed to the same extent when anxious or depressed participants were asked to judge the probability of the same events occurring to others. Depression has also been related to negative judgements in other domains, such as predictions about personal performance on intelligence tests or judgements of personal characteristics made by significant others [33]. Our current study extends this earlier work in two ways. First, we used bifactor modeling of questionnaire item-level responses to dissociate variance common to both anxiety and depression from that unique to anxiety or to depression. Second, we used computational modeling of judgements obtained within a carefully controlled, but naturalistic, belief updating task to disentangle the specific relationships between depression and anxiety respectively and negative belief priors and asymmetric belief updating.

Our first major finding was that elevated depression-specific affect was associated with increased negativity of ‘prior’ beliefs. In interpreting this result, it is important to consider whether it can simply be explained by information about other participants (e.g., being more skilled or desirable partners) that participants have picked up while doing the second ‘rating’ phase of the task. This does not seem likely—our findings indicate that participants are poor at estimating their own popularity, as potential internship partners, and that their ability to do this does not vary as a function of depression or anxiety specific affect; nor does actual ranking (i.e., popularity) mediate the relationship between depressed affect and the negativity of participants’ pre-feedback prior belief.

Alternatively, and more interestingly, participants might use pre-existing schema, or generalize from prior life events, to inform their time 0 beliefs. In relation to the former of these possibilities, classical theories of depression (see [5,34]) suggest that individuals with high levels of depression are more prone to using global stable negative self-referential beliefs to inform situational-specific assessments. This might well encompass using global negative self-referential beliefs about popularity to inform belief priors in novel situations such as that posed by this experiment. In relation to the latter possibility, negative priors may be informed by negative biases during the retrieval of related events from memory (e.g., past internships or peer evaluations). Indeed, a number of previous studies have linked depression to heightened recall of negative experiences ([35]; reviewed in [36]). Equally, negative priors might arise from biased simulation of possible alternatives if, prior to feedback, participants imagined the possible choices that other participants made in part 2 of the experiment. It has also been reported that individuals with elevated depression levels tend to generate fewer positive events than controls when asked to think about their personal future during a limited amount of time [1,37]. Both biases in recall and simulation can be conceptualized in terms of altered ‘model-based’ decision-making.

We note that each of possible mechanisms for the genesis of biased priors considered above may also reflect other influences operating much further back in time. Here, influential cognitive theories have proposed that many negative biases (in memory retrieval, simulation, interpretation etc.) reflect dysfunctional schemas, that were formed as a result of adverse life experiences in childhood [6,38].

Asymmetric integration of positively or negatively valenced feedback of pertinence to the current specific situation that an individual finds themselves within is another potential source of bias in beliefs. In the current experiment, we found that anxiety-specific affect was linked to a bias towards greater updating of beliefs following negative versus positive feedback. Previous research on belief updating largely comes from experiments asking participants to update their estimates for the probability of future life events occurring (e.g., getting cancer) using base rate information [10]. In such studies, depressed patients have been observed to be less optimistically biased than healthy controls, with patients with the highest levels of symptoms showing pessimistic updating [11,13]. In more recent work, negatively biased updating has been shown to depend on perceived threat and self-reported state anxiety [39]. In that work, optimistic updating was found to be abolished under threat, both in an experimental setting (using the Trier Social Stress Test) and in the field (in the case of firefighters on call).

One limitation of these prior studies is that they rely on participants making judgments about the probabilities of future life events before and after being given base rate information. As noted in the introduction, individuals may differ in exposure to life events that reasonably influence the perceived self-applicability of these base rates (e.g., if one has a prior history or family history of cancer), and this might potentially confound the estimation of biases in belief updating [15]. Another limitation is that few of these studies have been designed to disambiguate whether biases in belief updating are primarily linked to anxiety or depressive related symptomatology.

One question that arises from our findings is that, if individuals with high levels of anxiety-specific affect show a bias in updating, would we not expect them to also show a difference in prior beliefs as a result of previous asymmetric learning? The group-level positive relationship we found between updating and prior biases is consistent with an expectation that the former should ultimately lead to the latter, as subjects form priors from experience. Why then are negatively biased prior beliefs not associated with high levels of anxiety-specific affect, but only with high levels of depression-specific affect? One possibility is that individuals with high anxiety-specific affect might tend to use more situation-specific beliefs than individuals with high depression-specific affect and to engage in threat-monitoring. This would make them very sensitive to negative information, such as that provided by feedback in the current experiment. In contrast, individuals high in depression-specific affect might be more likely to generalize biased priors from negative global, stable, internal beliefs, particularly when these pertain to self-worth and competence [5,34,40,41]. That said, we did observe a non-significant trend towards a relationship between scores on the depression-specific latent factor and the interaction of the prior belief parameter and updating bias parameter, with depression-specific latent scores being elevated to the greatest extent in those individuals with both negative priors and negatively biased updating. Replication of the current work using a larger sample size would enable us to determine if this trend-level effect is simply a spurious result or indicative of a genuine interaction between bias in prior beliefs and asymmetric updating.

The hypothesis that elevated anxiety is linked to greater reliance on novel task-specific information to inform beliefs whereas elevated depression is linked to greater generalization from global beliefs when the current situation matches existing areas of concern (e.g., self worth) would also be empirically testable in future studies. In addition, it would be interesting for future work to investigate how beliefs change over the course of longer time scales. Individuals with elevated depression-specific affect might, for example, have their beliefs drift back to their initial negative state, even if they have been updated to a more neutral value during a particular situation. This could arise if they readily engage in biased offline processes (e.g., simulation, retrieval). It would also be important to conduct a similar experiment that targeted anxious schemas, perhaps by shifting the focus to something more akin to threat to see if the relationship with negativity of prior beliefs switches to anxiety and whether biases in updating are amplified.

Finally, we note that our participants were drawn from undergraduate students recruited through the Department of Psychology at UC Berkeley. The homogeneity of this sample helped with constructing a naturalistic task applicable to their career stage and experiences. However, a limitation is that this largely female well-educated sample is not representative of the population at large. Hence, in future work we would also hope to assess if our current findings generalize to other demographic groups.

In summary, we devised a novel, naturalistic task to explore the association between negative priors and negatively biased belief updating with different dimensions of Internalizing psychopathology. Using a bifactor analysis we decomposed Internalizing symptoms into three components—general negative affect, anxiety-specific affect and depression-specific affect. We found that elevated scores on the depression-specific factor, alone, were significantly associated with negativity of prior beliefs, whereas elevated scores on the anxiety-specific factor, alone, were significantly associated with negatively biased belief updating. This finding suggests that different processes lead to the negative, self-referential beliefs that characterize depression and anxiety. The association of elevated depression-specific affect with negative priors in the absence of updating biases might reflect greater reliance on, and bias in, model-based processes such as memory retrieval and simulation or on generalization from pre-existing schema. In contrast, anxiety-specific affect appears to be potentially associated with more negatively biased in-the-moment integration of information. This hypothesized differential reliance on generalization versus situation-specific learning in depression versus anxiety generates testable hypotheses for future research. More broadly, we hope that these findings will enhance our understanding of common versus distinct alterations to cognitive processes in anxiety and depression and, in the long-term, hopefully aid in the design of cognitive and behavioral interventions that are better aligned to an individual’s symptom profile.

## Methods

### Ethics statement

Informed written consent was obtained for all participants. Procedures were approved by the UC Berkeley committee for the protection of human subjects (Protocol ID: 2010-12-2638).

### Participants

Participants were undergraduates at UC Berkeley who enrolled in the study via the Psychology department’s Research Participation Program. Participants in part 1 (online profile generation) indicated if they wish to take part in part 2 (online rating of profile pairs) and part 3 (in-lab feedback session and judgement of one own’s popularity versus that of others). Parts 1 to 3 were spaced out to allow for all participants to complete a given part of the study before initiation of the next part of the study. Three recruitment rounds were conducted. Only interested participants who passed catch questions in part 1 were invited to take part in parts 2 and 3. In our second recruitment round, we lost a relatively large number of participants between parts 2 and 3 as the university campus was closed due to CA fires. Our final participant sample who completed all three parts of the study comprised 75 individuals (6 males; 69 females; age mean = 21.2, std. deviation = 2.1). Total numbers for part 1 were 349 and for part 2 were 145.

### Questionnaire measures

The full set of questionnaires administered included: the Penn-State Worry Questionnaire (PSWQ; [20]) the Mood and Anxiety Symptom Questionnaire (MASQ; [21,22]), the Spielberger State-Trait Anxiety Inventory (STAI form Y; [23]), and the Center for Epidemiologic Studies Depression Scale (CESD; [24]).

### Procedure

#### Part 1: online profile construction

A link to the Part 1 website was provided to students who signed up for the experiment through the Psychology Department’s research participant pool. Procedures were approved by the UC Berkeley committee for the protection of human subjects (Protocol ID: 2010-12-2638). Informed written consent was obtained and the task was to provide a profile for a ‘hypothetical internship’ described. Participants knew the internship was hypothetical, but that, if they chose to take part in Part 2, they and other students would be genuinely selecting among one another to choose teammates they thought would make optimal partners for such an internship project. It was explained that each participant would construct a personal profile to be used in this subsequent selection process. Many undergraduates at UC Berkeley do apply for highly competitive tech internships and piloting indicated that undergraduates were keen to make as competitive a profile as possible. For the profile, participants answered questions about their working style, their grades and SAT scores, and wrote a brief three sentence explanation about why they would make a good internship teammate. Participants were told that this information would be used to construct a profile for them that would be shown to the other participants. They were told that at the end of this part of the study, they could change their mind and chose not to continue on, in which case no-one else would see their profile. Participants also filled out the Penn-State Worry Questionnaire (PSWQ [20]) at the end of the session. They were informed that the answers to this questionnaire would not be shown to other participants.

#### Part 2: Online Profile comparison

Participants who wanted to take part in parts 2 and 3 of the experiment, and who correctly answered a single catch question embedded in the PSWQ, were sent an email inviting them to take part in the second online part of the experiment one to three weeks after their participation in part 1. In part 2, participants were presented 26 pairs of profiles from other participants who had also taken part in part 1. These profiles were anonymized by assigning them an ID number and removing information from the profile that clearly could have been used for identification (e.g., being captain of a particular sports team). Participants had to choose one of the two people in each profile pair that they’d prefer to work with during the hypothetical internship. During part 2, participants also filled out several more questionnaires tapping depression and anxiety symptomatology at the end of the session. These included the Mood and Anxiety Symptom Questionnaire (MASQ; [21,22]), the Spielberger State-Trait Anxiety Inventory (STAI; [23]), and the Center for Epidemiologic Studies Depression Scale (CESD; [24]). At the end of part two, participants marked a box indicating whether they were willing to be contacted for part three.

#### Part 3: In-lab feedback and probability ratings

Participants who wished to continue the study were invited to come into the UC Berkeley Department of Psychology to complete part three of the experiment. This final part was conducted between two and six weeks after part two. This time gap enabled us to obtain all part 2 ratings and to use these to select comparisons pairs to be shown to each participant. In part three, participants were first asked to rate the probability that they were in either (i) the most popular half of part 2 participants or (ii) the least popular half of part 2 participants (based on how often they were selected over another participant to be a hypothetical team partner). Question framing (least or most popular half) was counterbalanced across participants. Next, each participant was given twenty pieces of feedback. For each piece of feedback, participants were first shown their own profile along with a competitor’s profile and then shown which of the two profiles had been selected by a third participant, along with a statement such as *Classmate #29 compared you and classmate #14*. *You were chosen to work with*!. Participants were shown actual pairs of profiles and choices made by other participants. However, the pairs shown were chosen to provide balanced feedback. In each case, we selected 10 positive feedback instances, where the participant was chosen over the classmate and 10 negative feedback instances, where the classmate was chosen over the participant. All part 3 participants had at least 10 instances of each from which we could select exemplars. Negative and positive feedback items were presented in one of two predetermined sequences. After viewing the feedback for as long as they liked, participants then re-rated the probability that they were in either (i) the most popular half of part 2 participants or (ii) the least popular half of part 2 participants after each piece of feedback. Participants spent around 5 ±2 seconds per profile pair (median: 5.41s; 25th-percentile: 3.56s; 75th-percentile: 7.57s). Following these self-referential judgements, participants were asked to complete a parallel task involving other-referent ratings. Here, the same procedure was used except that participants were shown the profile for another participant (labelled by a pseudoID) and asked to judge the probability that this other participant was in the top/bottom half both prior to feedback and after presentation of each of 20 pieces of feedback pertaining to how this other participant was compared against others. At the end of the session, participants re-completed the STAI and CESD questionnaires.

### Data exploration: data quality checks and data-set exclusion

We sought to exclude participants who showed unusual or outlying behavior potentially indicative of poor understanding of the task or lack of concentration. To address this, we examined whether participants updated their beliefs in the opposite direction to that which would be suggested by the feedback. Rational behavior would be to either keep beliefs unchanged or to change them in the direction suggested by the feedback. 48 participants (64%) made a directionally wrong update on 2 trials or fewer. Another 25 participants (33%) made between 3 and 8 directional errors. Three participants (4%) made 9 or more directional errors–datasets for these three participants were excluded. A fourth participant was excluded because he reported misunderstanding the task in the post-experiment interview.

To check whether participants were paying attention to the self-report questionnaires, we looked at ‘catch questions’. Catch questions instructed the participant to select a particular response (e.g., ‘sometimes’) from the set of available responses (e.g., ‘never’, ‘sometimes’, ‘almost always’, ‘always’). Of the seventy-five participants who completed all three sessions, four participants were also excluded for failing to correctly answer 50% or more of the “catch questions”. There were four catch questions in total, one from the PSWQ (session 1), two from the MASQ (session 2), and one from the STAI (session 3). In order for participants to be invited for session 2, they had to correctly answer the PSWQ catch question from session 1. The number of participants who did not correctly complete this question are reported above in the ‘Participants’ section. The four participants considered here, who were excluded at the end of session 3, answered the first catch question correctly, but then answered two out of the three subsequent catch questions incorrectly. An additional participant chose the same response option for all items on the MASQ questionnaire. This left a total of 66 participants whose data we analyzed in detail.

### Calculating participants’ scores on latent dimensions of anxiety and depression specific affect and general negative affect

We previously conducted a bifactor analysis of responses to questionnaire items taken from the STAI, the CESD, the BDI, the MASQ anxious arousal subscale, the MASQ anhedonic depression subscales, the PSWQ, and the EPQ Neuroticism subscale [25]. This was conducted in a sample of eighty-six participants comprised of patients diagnosed with major depressive disorder (MDD) or generalized anxiety disorder (GAD), healthy control participants screened to be free of any psychiatric diagnoses, and a community sample with naturally varying levels of symptoms. The loadings of individual questionnaire items on a general negative affect factor and specific Anxiety and Depression factors were estimated. The general factor had high loadings (>0.4) for multiple anxiety-related and depression-related items and moderately high loadings (>0.2) across almost all items. The depression-specific factor had high loadings (>0.4) for questions related to anhedonia and depressed mood. The anxiety-specific factor had high loadings (>0.4) for questions related to worry and anxiety. This factor structure was similar to that in previously reported by studies using bifactor analyses of anxiety and depression related symptomatology [42–46]. The factor structure was validated by conducting a confirmatory bifactor analysis in an independent online sample of participants (n = 199) comprised of students at UC Berkeley (120 females, mean age = 20 ± 4), which has a similar demographic make-up and was recruited in an equivalent fashion to participants in the current study.

The factor loadings matrix from this prior work was used to calculate factor scores for participants in the current study. To do so, we regressed current participants’ responses onto the previously estimated factor loadings. We used the Anderson-Rubin method [47] to preserve orthogonality while calculating the scores. This was done using the “factor.scores” function in R. Neither the BDI nor the EPQ were administered in the current study, so the loadings on items from these questionnaires were omitted from this regression procedure. To check the validity of using this reduced set of items, we calculated factor scores for the participants in Gagne et al (2020) [25] using this reduced item set (n = 95 items) and correlated these scores with those obtained using the full set of items (n = 128 items). For each of the three factors, scores obtained using the full and reduced set of item loadings were highly correlated (rs> = 0.98).

Participants in the current study completed the STAI and the CESD in two different sessions; the responses in each session were concatenated prior to calculating the factor scores. We note that averaging the scores across sessions yielded extremely similar factor scores (rs>0.98). Administering the STAI and CESD in two different sessions also allowed us to calculate test-retest reliability. The test-retest reliability for STAI was very good (r(64) = 0.91), whereas it was more moderate for CESD (r(64) = 0.53). The CESD test-retest reliability varied widely across individual items, ranging from r(64) = 0.69 for “I could not get going” to r(64) = 0.2 for “my appetite was poor”.

### Computational Models: The four main models

#### Models with Bayesian updating with (model 1) and without (model 2) a parameter to capture biased updating

Participants’ beliefs, which corresponded to the probability that they were in the top half (or bottom half) of participants, were modeled by a Beta distribution *B*(*α*_*t*_, *β*_*t*_). Using a whole distribution allowed us to model both a point estimate (for instance, the mean) that the participants might have entertained and the uncertainty (e.g., the variance) about that estimate. The two parameters *α*_*t*_, *β*_*t*_ of the Beta distribution can be interpreted as the weight of evidence for or against the probability of being in the top-half of the participants, respectively, and their sum can be interpreted as the total degree of certainty in their belief. We considered participants’ actual reports, ${\hat{q}}_{t}∼B(\alpha_{t},\beta_{t})$, at any time point *t* to be a single sample from their Beta distribution at that point. This means that participants with high degrees of certainty would report beliefs tightly centered around the mean of their distribution, while those less certain would report a wider range of beliefs.

The Bayesian update models (Models 1 and 2) start from a prior distribution, *B*(*α*_0_, *β*_0_). The parameters *α*_0_, *β*_0_ are estimated separately for each participant and characterize the participant’s prior belief before the start of the feedback period (i.e., at *t* = 0); the mean of this prior distribution is given by for $\frac{\alpha_{0}}{{(\alpha }_{0}+\beta_{0})}$. The values for *α*_*t*_, *β*_*t*_ for *t*≥1 are then updated based on feedback on trial *t*; this feedback is either positive, *X*_*t*_ = 1, or negative, *X*_*t*_ = 0. In model 1, updating includes the effect of a bias parameter (*ω*; also estimated per participant) which allows for different rates of updating following positive versus negative feedback. Negative and positive biases are given by *ω*<1 and *ω*>1, respectively. In model 2, the bias parameter (*ω* = 1) is fixed to one, forcing updating to be unbiased. In both cases, the update is given by Eq 2.


$$\alpha_{t}=\alpha_{t-1}+\omega X_{t}$$

$$\beta_{t}=\beta_{t-1}+\frac{1}{\omega }(1-X_{t})$$ (2)

For the unbiased (‘pure’ Bayesian) model (model 2), Eq 2 is exactly integrating prior expectations (*α*_*t*_, *β*_*t*_) with likelihood information (the feedback) to generate a posterior (*α*_*t*+1_, *β*_*t*+1_), which then becomes the prior for the next trial. For the biased Bayesian model (model 1), this update process is approximate. For both models, the updates given by Eq 2 produces distributions of belief that become narrower as information is progressively incorporated, and so the effective size of the updates reduces over trials.

In summary, models 1 and 2 have three and two free parameters, respectively: (model 1: *α*_0_∈[2, 100], *β*_0_∈[2, 100], *ω*∈[0, 5]; model 2: *α*_0_∈[2, 100], *β*_0_∈[2, 100]).

#### Models with RW updating with (model 3) and without (model4) a parameter to capture biased updating

Identically to the Bayesian model, the Rescorla-Wagner models (Models 3 and 4) also model a participant’s reported belief on trial *t*≥0 as a single sample from a Beta distribution ${\hat{q}}_{t}∼B(\alpha_{t},\beta_{t})$. However, in contrast to the Bayesian models, this distribution is alternatively parameterized according to its mean (*μ*_*t*_) and a constant precision (*ν*; i.e., belief certainty); these relate to the standard parameters through: *α*_*t*_ = *νμ*_*t*_; *β*_*t*_ = *ν*(1−*μ*_*t*_). The mean starts at a value *μ*_0_ (i.e. prior belief), which is estimated separately for each participant. It is then updated directly in response to feedback (*X*_*t*_) using a learning rate (*η*; also estimated per participant). In model 3, updating is scaled by a bias term (*b*; again participant-specific), with negative and positive biases given by *b*<1 and *b*>1, respectively. To prevent the values for the mean from becoming greater than 1, an additional constraint is imposed on the mean during parameter estimation. In model 4, the bias parameter (*b* = 1) is fixed to one, forcing updating to be unbiased. In both cases, the update is given by Eq 1 (Results section; reproduced below for convenience).


$$\mu_{t}={min(\mu }_{t-1}+\eta ({bX}_{t}-\mu_{t-1}),1)$$ (1)

In contrast to the Bayesian models, the update given by Eq 1 does not necessarily produce narrowing distributions of belief over time. As a result, if the Bayesian and RW models started with equivalent update sizes, feedback later on in the experiment indicating a need to revise beliefs would result in slower revision of beliefs by the Bayesian models relative to the RW models.

In summary, models 3 and 4 have four and three free parameters, respectively: (model 3: *μ*_0_∈[0,1], *ν*∈[2,1000], *η*∈[0,1], *b*∈[0,5]; model 4: *μ*_0_∈[0,1], *ν*∈[2,1000], *η*∈[0,1]).

### Model parameter estimation

To prevent outliers during parameter estimation, we used Maximum a Posteriori (MAP) estimation rather than maximum likelihood estimation. Priors were assigned to each parameter, and the mode of the posterior was used as the estimate. For the Bayesian belief updating models, *α*_0_ and *β*_0_ were assigned a standard, uninformative HalfNormal prior (mean = 0, sd = 50). We additionally constrained these two parameters to be in the following range: [2, 100]. The bias parameter *ω* was assigned a Normal prior (mean = 1, sd = 0.5), and was also constrained to be in the following range: [0, 5]. For the RW models, the precision parameter *ν* was assigned a HalfNormal prior (mean = 0, sd = 500) and constrained to be in the following range: [2, 1000]. The initial mean of the belief distribution *μ*_0_ was assigned a Normal prior(mean = 0.5, sd = 0.5) and constrained to be in the following range: [0, 1]. The learning rate parameter *η* was assigned a HalfNormal prior (mean = 0, sd = 0.5) and constrained to be in the following range: [0, 1]. The bias parameter *b* was assigned a HalfNormal prior(mean = 0, sd = 2) and constrained to be in the following range: [0, 5].

### Model comparison

The four main models were compared using exceedance probabilities, which are defined as the probability that a given model is the most prevalent at the population level. Exceedance probabilities are estimated using hierarchical Bayesian inference [29]. This requires an estimate of log model evidence; for this we used the BIC. The BIC for each participant for each model was calculated using the MAP parameter estimates as follows: BIC = $-2*log\;(y|{\hat{\theta }}_{MAP})+k*log\;(n)$ (where k is the number of parameters and n = 20 for the number of reported beliefs per participant).

In the supplemental materials, we conduct the same model comparison procedure on a larger set of models, which includes nine additional models. For more details see S2 Text.

### Correlation analyses and FDR correction for multiple comparisons

To account for the possibility that the parameter values might be non-normally distributed, we used permutation-based tests to calculate the significance of the correlations between parameter values and scores on the three latent symptom factors in Figs 2 and S5. The data were randomly permuted and the correlation coefficient *r* was calculated for each permuted dataset; this was repeated 10,000 times to create the null distribution for the correlation coefficients. The statistical significance of the actual correlation coefficient (i.e., that estimated from the non-permuted data) was calculated as the percentage of samples from the null distribution more extreme (or less extreme in the case of negative values) than actual correlation coefficient; for a two-tailed test, the distribution and the coefficient were transformed using the absolute value.

When reporting correlations between the final model parameters (Model 3) and the factor scores, we corrected the p-values for multiple comparisons. We corrected for twelve tests (the three symptom factors by the four model parameters). The correction for multiple comparisons was done using the Benjamini-Hochberg procedure [48].

## Supporting information

S1 Text Supplemental Model-Agnostic Analyses. To complement the model-based analyses presented in the main text, model-agnostic methods were used to examine biases in prior beliefs and belief updating at a group level and as a function of latent dimensions of anxiety, depression and general negative affect.
(DOCX)

S2 Text Supplemental Model-Based Analyses. Here we present supplementary model-based analyses including analyses of the effect of feedback order and detailed description of supplementary models against which the winning model was also compared.
(DOCX)

S1 Table A comparison of participants’ anxiety and depression summary scores with those for individuals diagnosed with MDD and GAD as well as healthy controls and an unselected community sample (as reported by [25]). Summary scores for each of the questionnaires administered are shown for participants in the current study as well as for those from a prior study [25], which included individuals with MDD, GAD, healthy controls and an unselected community sample (columns 2 to 5, respectively). In addition to giving the mean and SD for scores on each measure for each group, we also report the number of our current participants who fell within one standard deviation of the mean for the MDD and GAD groups, see square brackets and note 2. STAI = Spielberger State-Trait Anxiety Inventory (form Y; [23]); MASQ-AD/MASQ-AA = anhedonic depression and anxious arousal subscales for the Mood and Anxiety Symptoms Questionnaire [21,22]; PSWQ = Penn State Worry Questionnaire [20]; CESD = Center for Epidemiologic Studies Depression Scale [24].
(DOCX)

S2 Table Model Agnostic Analysis Results. Participants’ standardized scores for the general factor, anxiety-specific factor and depression-specific factor (columns) are correlated against model agnostic measures of prior beliefs and belief updating (rows). Participants reported believes about themselves (‘self’; first three rows) and for another randomly chosen participant (‘other’; last three rows). Pearson correlations were used to examine the relationship between scores on the three latent symptom factors and our two model agnostic indices of interest. Here uncorrected p values are given, p values that survive multiple comparison correction at p < .05 are indexed by *.
(DOCX)

S3 Table Regression models that use parameter estimates for prior mean (*μ*_0_) and bias in belief updating (*b*) for self-referential data to predict scores on the depression-specific factor, the anxiety-specific factor and general ‘negative affect’ factor. Each regression analysis uses scores on one of the three latent factor dimensions as the dependent variable and parameter estimates for both *μ*_0_ and *b* as predictors. Parameter estimates were obtained using the winning model: Model 3 the “biased RW” model. These regression analyses confirmed our original finding of a significant relationship between *μ*_0_ and depression-specific affect and a significant relationship between *b* and anxiety-specific affect for self-referential judgements.
(DOCX)

S4 Table Regression models that use parameter estimates for prior mean (*μ*_0_) and bias in belief updating (*b*) for other-referent data to predict scores on the depression-specific factor, the anxiety-specific factor and general ‘negative affect’ factor. Each regression analysis uses scores on one of the three latent factor dimensions as the dependent variable and parameter estimates for both *μ*_0_ and *b* as predictors. Parameter estimates were obtained using the winning model: Model 3 the “biased RW” model. These additional regressions confirmed the findings from our correlational analyses, with neither *μ*_0_ or *b* significantly predicting scores on any of the three latent dimensions, and with only a trend-level relationship between other-belief *μ*_0_ and depression-specific affect, (t = -1.870, p = 0.066).
(DOCX)

S1 Fig Correlation of scores on the three latent factors and questionnaire subscales, across participants. Factor loadings from a previously published bifactor analysis of item-level responses to self-report questionnaire measures of anxiety and depression [25] was used to estimate current participants’ scores on three orthogonal latent factors: a general Negative Affect factor and Anxiety and Depression specific factors. To check the concurrent validity of these factors in the current sample, we correlated scores on each factor (y axis) with scores on each of the subscales administered (x axis). Scores on the general factor showed a positive correlation with scores across all of the subscales. Scores on the depression-specific factor correlated most strongly with scores on the anhedonia-related subscales (e.g., MASQ anhedonia; CESD anhedonia). Scores on the anxiety-specific factor correlated most strongly with scores on the PSWQ. Self-report questionnaire measures were administered at the end of one or more sessions; for details, see Methods.
(TIF)

S2 Fig Model reproduction of average belief updates following positive versus negative feedback. The four models considered were each used to simulate 20 new estimated judgements for each participant for each trial, using that participant’s parameter estimates for the model in question. Here, we plot the trial-to-trial changes in judgments, averaged separately for trials following positive (x axis) versus negative (y axis) feedback, for both the simulated data (in black) and the original data (in blue). Each panel shows the results for a separate model. These models differ along two dimensions: (i) whether beliefs are updated either according to Bayesian or Rescorla-Wagner (RW) principles (separated by column), and (ii) whether a bias in updating after negative versus positive feedback was incorporated (separated by row). It can be seen that simulated data from Model 3, ‘the biased RW model’ (top right panel) provides a better match to the distribution of participants’ updates than is the case for the other three models considered; a Kolmogorov-Smirnov test confirms that there are significant differences between the simulated and real data distributions for models 1, 2 and 4 (p<0.001) but not for model 3 (p = 0.5). Note, positive feedback should lead to a positively signed update and negative feedback to a negatively signed update, hence data points in all but the bottom right quadrant reflect updating in the opposite direction to the feedback provided. Note further that beliefs in the model are sampled, and so need not normatively occupy that quadrant.
(TIF)

S3 Fig Exceedance probability-based model comparison. Models were compared using exceedance probability, which is defined as the probability that a model is the most prevalent at the population level. Exceedance probabilities are estimated using hierarchical Bayesian inference [29]. The biased RW model (Model 3) was estimated to be the most prevalent (most likely) model, with an exceedance probability of 0.67. The second most prevalent model was Model 10, a biased Bayesian model with separate reporting and updating distributions. This model had a much lower exceedance probability of 0.25. For descriptions of each model, see S2 Text.
(TIF)

S4 Fig Confusion matrix for model recovery simulations. Each of the models on the y-axis were considered, in turn, to be the true model and used to generate data for 100 new participants; new parameter values were chosen for each simulated participant by sampling from distributions informed by the distribution of actual parameter estimates. The simulated data were fit by each of the possible models listed on the x-axis and the percentage of participants for which each ‘recovery’ model had the best penalized fit (BIC) is shown in the cells along each row. The percentages add up to 100% for each simulated dataset (i.e., across each row). This was completed for all the generative models on the y-axis. A high percentage along the diagonal indicates high model recoverability—i.e., that the model that generated the data was chosen as the best fitting model against all others. Models 1 to 4 correspond to the four main models presented in the main manuscript. Models 5–13 correspond are presented in S2 Text, Supplemental Model-based Analyses: Additional models and model comparison.
(TIF)

S5 Fig Correlations between model parameter estimates for learning rate (*η*) and reporting precision (*ν*) and scores on the depression-specific and anxiety-specific latent factors. Participants’ standardized scores for the general factor, anxiety-specific factor and depression-specific factor (x axes) are plotted against the two other parameters in the main model (Model 3: biased RW). *η* is the estimated learning rate, which measures the absolute amount that beliefs are updated in response to feedback, and *ν* is the estimated precision of the reporting belief distribution, which measures how close participants’ reported beliefs are to the mean of their belief distribution. Neither *η* nor *ν* significantly correlated with scores on any of the three Internalizing symptom factors (uncorrected p-values shown here). After removing the outlier for learning rate (outlined in red), the correlation between *η* and scores on the anxiety-specific factor rose to r(64) = 0.25 (uncorrected p = 0.048), but this does not survive correction for multiple comparisons (corrected p = 0.19).
(TIF)

S6 Fig Exceedance probability-based model comparison for ‘other-referent’ beliefs. Participants were also asked to report and updating beliefs about another randomly chosen participant. The same thirteen models were fit to these other-referent belief data and compared using exceedance probability. The biased RW model (Model 3) was again estimated to be the most prevalent (most likely) model, with an exceedance probability of 0.62. This model comparison result closely match that for the self-referent data (S3 Fig).
(TIF)

S7 Fig Correlations of model-agnostic starting belief, ending belief, belief change (ending–starting belief) with individual questionnaire subscales. P-values are shown in parentheses underneath Pearson correlation values. In line with the factor score results, subscales tapping anhedonic depression were negatively correlated with starting belief (CESD anhedonia, MASQ anhedonia, STAI depression). These model-agnostic measures of belief updating and broad-brush measures of anxiety and depressive symptomatology, that do not dissociate variance common to both anxiety and depression from that unique to anxiety or depression, do not provide a clear picture of a relationship between negative affect and bias in belief-updating.
(TIF)

S8 Fig Relationship between model estimated starting and ending beliefs, split by feedback order. Participants beliefs, as estimated by the model, are plotted for the start and for the end of the feedback period. Participants are split into two groups based on which feedback sequence they received: the positive-first feedback sequence, which started with positive feedback for the first two trials and had a total of six positive feedbacks in the first ten trials, or the negative-first feedback sequence, which was exactly the opposite. In both groups, participants update towards a neutral belief (i.e., 50%) after receiving twenty instances of balanced feedback. This partial updating towards 50% is indicated by the two regression lines, both which have a slope (0.49 for positive-first; 0.74 for negative-first) significantly greater than 0 and significantly less than 1. However, participants who received positive feedback first shifted their beliefs more in the positive direction from start to end (independent t-test, difference between groups for ending-starting beliefs, t(65) = 4.48, p < 0.001).
(TIF)

S9 Fig Best and Worst Model Fits. Participants in which model #3 fits best (left column) and worst (right column) are shown. The black line is the participant’s reported belief for self-referential judgments and the blue line is the model #3’s posterior mean prediction; error bars represent ±1 posterior standard deviation. The left columns shows that the model can fit extremely well (R-squared >0.93) for a variety of different of behaviors. The right column shows examples of behavior that cannot be captured, such as early updating followed by no updating (e.g., participant #41) or participants with highly variable magnitudes in updating (participant #58). Note that these two participants are not better fit by the second best model (model #10), with lower R-squared in all four cases.
(TIF)

## References

[1] MacLeod AK, Byrne A. Anxiety, depression, and the anticipation of future positive and negative experiences. J Abnorm Psychol. 1996;105: 286. doi: 10.1037//0021-843x.105.2.286 8723011

[2] MacLeod AK. Affect, emotional disorder, and future-directed thinking. Cogn Emot. 1996;10: 69–86.

[3] Butler G, Mathews A. Cognitive processes in anxiety. Adv Behav Res Ther. 1983;5: 51–62.

[4] Muris P, van der Heiden S. Anxiety, depression, and judgments about the probability of future negative and positive events in children. J Anxiety Disord. 2006;20: 252–261. doi: 10.1016/j.janxdis.2004.12.001 16464708

[5] Beck AT. Depression Harper and Row: New York. 1967.

[6] Beck AT. Cognitive therapy and the emotional disorders. Penguin; 1979.

[7] Abramson LY, Metalsky GI, Alloy LB. Hopelessness depression: A theory-based subtype of depression. Psychol Rev. 1989;96: 358.

[8] Frank MJ, Moustafa AA, Haughey HM, Curran T, Hutchison KE. Genetic triple dissociation reveals multiple roles for dopamine in reinforcement learning. Proc Natl Acad Sci. 2007;104: 16311–16316. doi: 10.1073/pnas.0706111104 17913879 PMC2042203

[9] Palminteri S, Pessiglione M. Opponent brain systems for reward and punishment learning: causal evidence from drug and lesion studies in humans. Decision Neuroscience. Elsevier; 2017. pp. 291–303.

[10] Sharot T. The optimism bias. Curr Biol. 2011;21: R941–R945. doi: 10.1016/j.cub.2011.10.030 22153158

[11] Garrett N, Sharot T. How robust is the optimistic update bias for estimating self-risk and population base rates? PLoS One. 2014;9: e98848. doi: 10.1371/journal.pone.0098848 24914643 PMC4051586

[12] Chowdhury R, Sharot T, Wolfe T, Düzel E, Dolan RJ. Optimistic update bias increases in older age. Psychol Med. 2014;44: 2003–2012. doi: 10.1017/S0033291713002602 24180676 PMC4035755

[13] Korn CW, Sharot T, Walter H, Heekeren HR, Dolan RJ. Depression is related to an absence of optimistically biased belief updating about future life events. Psychol Med. 2014;44: 579–592. doi: 10.1017/S0033291713001074 23672737 PMC3880066

[14] Sharot T, Kanai R, Marston D, Korn CW, Rees G, Dolan RJ. Selectively altering belief formation in the human brain. Proc Natl Acad Sci. 2012;109: 17058–17062. doi: 10.1073/pnas.1205828109 23011798 PMC3479523

[15] Shah P, Harris AJ, Bird G, Catmur C, Hahn U. A pessimistic view of optimistic belief updating. Cognit Psychol. 2016;90: 71–127. doi: 10.1016/j.cogpsych.2016.05.004 27542765

[16] Stankevicius A, Huys QJ, Kalra A, Seriès P. Optimism as a prior belief about the probability of future reward. PLoS Comput Biol. 2014;10: e1003605. doi: 10.1371/journal.pcbi.1003605 24853098 PMC4031045

[17] Dobson K, Franche R-L. A conceptual and empirical review of the depressive realism hypothesis. Can J Behav Sci Can Sci Comport. 1989;21: 419.

[18] Eil D, Rao JM. The good news-bad news effect: asymmetric processing of objective information about yourself. Am Econ J Microecon. 2011;3: 114–38.

[19] Möbius MM, Niederle M, Niehaus UP. Managing self confidence: Theory and experimental evidence. NBER Working Paper 17014. 2011.

[20] Meyer TJ, Miller ML, Metzger RL, Borkovec TD. Development and validation of the penn state worry questionnaire. Behav Res Ther. 1990;28: 487–495. doi: 10.1016/0005-7967(90)90135-6 2076086

[21] Watson D, Clark LA. Mood and anxiety symptom questionnaire. J Behav Ther Exp Psychiatry. 1991.

[22] Clark LA, Watson D. The mini mood and anxiety symptom questionnaire (Mini-MASQ). Unpubl Manuscr Univ Iowa. 1995.

[23] Spielberger CD. State-trait anxiety inventory for adults. 1983.

[24] Radloff LS. The CES-D scale: A self-report depression scale for research in the general population. Appl Psychol Meas. 1977;1: 385–401.

[25] Gagne C, Zika O, Dayan P, Bishop SJ. Impaired adaptation of learning to contingency volatility in internalizing psychopathology. Elife. 2020;9: e61387. doi: 10.7554/eLife.61387 33350387 PMC7755392

[26] Stewart N, Chater N, Brown GD. Decision by sampling. Cognit Psychol. 2006;53: 1–26. doi: 10.1016/j.cogpsych.2005.10.003 16438947

[27] Vul E, Goodman N, Griffiths TL, Tenenbaum JB. One and done? Optimal decisions from very few samples. Cogn Sci. 2014;38: 599–637. doi: 10.1111/cogs.12101 24467492

[28] Moutoussis M, Dolan RJ, Dayan P. How people use social information to find out what to want in the paradigmatic case of inter-temporal preferences. PLoS Comput Biol. 2016;12: e1004965. doi: 10.1371/journal.pcbi.1004965 27447491 PMC4957786

[29] Stephan KE, Penny WD, Daunizeau J, Moran RJ, Friston KJ. Bayesian model selection for group studies. Neuroimage. 2009;46: 1004–1017. doi: 10.1016/j.neuroimage.2009.03.025 19306932 PMC2703732

[30] Svenson O. Are we all less risky and more skillful than our fellow drivers? Acta Psychol (Amst). 1981;47: 143–148.

[31] Alloy LB, Abramson LY. Judgment of contingency in depressed and nondepressed students: Sadder but wiser? J Exp Psychol Gen. 1979;108: 441. doi: 10.1037//0096-3445.108.4.441 528910

[32] Tingley D, Yamamoto T, Hirose K, Keele L, Imai K. Mediation: R package for causal mediation analysis. 2014.

[33] Strunk DR, Adler AD. Cognitive biases in three prediction tasks: A test of the cognitive model of depression. Behav Res Ther. 2009;47: 34–40. doi: 10.1016/j.brat.2008.10.008 19010460

[34] Abramson LY, Seligman ME, Teasdale JD. Learned helplessness in humans: critique and reformulation. J Abnorm Psychol. 1978;87: 49. 649856

[35] Teasdale JD, Taylor R, Fogarty SJ. Effects of induced elation-depression on the accessibility of memories of happy and unhappy experiences. Behav Res Ther. 1980;18: 339–346. doi: 10.1016/0005-7967(80)90093-5 7436981

[36] Mathews A, MacLeod C. Cognitive vulnerability to emotional disorders. Annu Rev Clin Psychol. 2005;1: 167–195. doi: 10.1146/annurev.clinpsy.1.102803.143916 17716086

[37] MacLeod AK, Salaminiou E. Reduced positive future-thinking in depression: Cognitive and affective factors. Cogn Emot. 2001;15: 99–107.

[38] Clark DA, Beck AT. Cognitive theory and therapy of anxiety and depression: Convergence with neurobiological findings. Trends Cogn Sci. 2010;14: 418–424. doi: 10.1016/j.tics.2010.06.007 20655801

[39] Garrett N, González-Garzón AM, Foulkes L, Levita L, Sharot T. Updating beliefs under perceived threat. J Neurosci. 2018;38: 7901–7911. doi: 10.1523/JNEUROSCI.0716-18.2018 30082420 PMC6125815

[40] Butkowsky IS, Willows DM. Cognitive-motivational characteristics of children varying in reading ability: evidence for learned helplessness in poor readers. J Educ Psychol. 1980;72: 408. 7391337

[41] Sweeney PD, Anderson K, Bailey S. Attributional style in depression: a meta-analytic review. J Pers Soc Psychol. 1986;50: 974. doi: 10.1037//0022-3514.50.5.974 3712233

[42] Clark DA, Steer RA, Beck AT. Common and specific dimensions of self-reported anxiety and depression: implications for the cognitive and tripartite models. J Abnorm Psychol. 1994;103: 645. 7822565

[43] Steer RA, Clark DA, Beck AT, Ranieri WF. Common and specific dimensions of self-reported anxiety and depression: a replication. J Abnorm Psychol. 1995;104: 542. doi: 10.1037//0021-843x.104.3.542 7673579

[44] Simms LJ, Grös DF, Watson D, O’Hara MW. Parsing the general and specific components of depression and anxiety with bifactor modeling. Depress Anxiety. 2008;25: E34–E46. doi: 10.1002/da.20432 18027844

[45] Steer RA, Clark DA, Kumar G, Beck AT. Common and specific dimensions of self-reported anxiety and depression in adolescent outpatients. J Psychopathol Behav Assess. 2008;30: 163–170.

[46] Brodbeck J, Abbott RA, Goodyer IM, Croudace TJ. General and specific components of depression and anxiety in an adolescent population. BMC Psychiatry. 2011;11: 1–12. 22151586 10.1186/1471-244X-11-191 PMC3266209

[47] Anderson TW, Rubin H. Proceedings of the Third Berkeley Symposium on Mathematical Statistics and Probability: Held at the Statistical Laboratory, University of California, December, 1954, July and August, 1955. Univ of California Press; 1956. p. 111. s

[48] Benjamini Y, Hochberg Y. Controlling the false discovery rate: a practical and powerful approach to multiple testing. J R Stat Soc Ser B Methodol. 1995;57: 289–300.

