# Supplementary material for: Biased belief priors versus biased belief updating: Differential correlates of depression and anxiety
Source: PLoS Comput Biol. 2022 Aug 15;18(8):e1010176. doi: 10.1371/journal.pcbi.1010176 (PMC9377597; doi:10.1371/journal.pcbi.1010176)
Supplement: S1 Text — To complement the model-based analyses presented in the main text, model-agnostic methods were used to examine biases in prior beliefs and belief updating at a group level and as a function of latent dimensions of anxiety, depression and general negative affect. (DOCX) [file pcbi.1010176.s001.docx]

### Supplemental Model-Agnostic Analyses

#### Biases in prior beliefs: group level analyses

As a model agnostic index of participants’ starting, or ‘prior’ belief, we used their initial estimate of the probability that they were in the more popular half of students, as potential internship partners. In line with the model-based analyses, these estimates showed no evidence for a systematic optimism bias for self-referent beliefs (mean probability of being in top half = 0.49, t(65)= -0.33, p>.7), and did show evidence for one for other-referent beliefs, mean probability = 0.54, t(65)=2.59, p=0.01. However, the paired t-test for the difference between self versus other initial beliefs was only marginally significant, t(64)=-1.82, p=0.07. For both self- and other-referent beliefs there was no effect of framing on starting beliefs, t(64) = 0.25, p=0.8; t(64) = -0.39, p=0.69.

#### Biases in prior beliefs: relationship with Internalizing symptoms

Confirming the model-based results, scores on the depression-specific factor were significantly negatively correlated with starting belief, r(64) = -0.36, p = 0.003, FDR pcorr=0.03, corrected for all model-agnostic comparisons in S2 Table; and there was no effect of framing on this relationship, β=0.006, p>.9. The other-referent starting beliefs were not correlated with the depression specific factor, r(64)=-0.14, p=0.25. However, the self versus other difference did not reach significance, r(64) = -0.22, p=0.076. Neither scores for the anxiety-specific factor nor for the general negative affect factor correlated with starting beliefs (ps>0.4, S2 Table); there was also no significant interaction of scores on these factors and framing on initial beliefs, ps>0.7.

#### Belief updating: group level analyses

As a model-agnostic measure of belief updating, we used the difference in participants’ beliefs from the start to the end of the feedback period. There was no evidence of biased updating at the group-level for self-referent beliefs; across participants, the mean difference between starting and ending beliefs did not differ significantly from zero, mean=0.003; t=0.015; p=0.98. There was also no evidence of a group-level bias for other-referent beliefs, mean difference between starting and ending beliefs= 0.006, t=0.31, p=0.75. This latter (null) result contrasts with the model-based analysis, which showed a small but significant positivity bias (i.e., a mean $b$ = 1.16). This again may speak to an increased sensitivity of using model-based analyses over model-agnostic analyses; here, the model-agnostic estimates for bias rely on the difference between two point estimates, one at the start and one at the end of the feedback period, and are highly dependent on their accuracy.

#### Biases in belief updating: relationship with Internalizing symptoms

In partial agreement with the model-based results, there was a trend towards a negative relationship between scores on the anxiety-specific factor and change in beliefs from start to end of the feedback period, however it did not reach statistical significance: r(64) = -0.23, p = 0.065 uncorrected; S2 Table. Again, neither scores on the depression specific factor nor scores on the general factor were significantly correlated with the difference between end belief and starting belief (r(64) = 0.04, p = 0.77; r(64) = 0.13, p = 0.28; S2 Table). For other-referent beliefs, none of the three factors correlated with the difference between end and starting belief (anxiety, r(64) = -0.09, p = 0.48; depression r(64) = 0.06, p = 0.65; r(64) = -0.11, p = 0.38).

#### Beliefs at the end of the feedback period

At the end of all feedback, both depression-specific and anxiety-specific factor scores were negatively correlated with participants’ probability estimates of being in the most popular half, however neither of these relationships survived correction for multiple comparisons (r(64) = -0.26, p = 0.034 uncorr., r(64) = -0.24, p = 0.049, uncorr., respectively). There was no significant relationship between general negative affect scores and ‘end beliefs’, r(64) = 0.12, p = 0.33; S2 Table. Feedback order did not interact significantly with scores on any of the three symptom factors on either the difference between end and starting beliefs or end beliefs (ps>0.6). For other referent beliefs, there were no significant correlations between the end beliefs and the Internalizing factor scores (S2 Table).

#### Post-hoc correlations between model-agnostic measures and individual questionnaires for Internalizing symptoms

Post-hoc correlations with individual questionnaire scales are reported here to aid comparison across studies (see S7 Fig). These correlations are as expected given the depression-specific factor’s subscale correlations reported above, namely negative starting beliefs were predominantly linked to anhedonic symptomatology, as measured by the anhedonic subscale of the Mood and Anxiety Symptom Questionnaire (MASQ) and the anhedonic subscale of the of the Center for Epidemiologic Studies Depression Scale (CESD).
