# Supplementary material for: Biased belief priors versus biased belief updating: Differential correlates of depression and anxiety
Source: PLoS Comput Biol. 2022 Aug 15;18(8):e1010176. doi: 10.1371/journal.pcbi.1010176 (PMC9377597; doi:10.1371/journal.pcbi.1010176)
Supplement: S2 Text — Here we present supplementary model-based analyses including analyses of the effect of feedback order and detailed description of supplementary models against which the winning model was also compared. (DOCX) [file pcbi.1010176.s002.docx]

### Supplemental Model-based Analyses

#### Supplemental Analysis on the Effects of Feedback Order

Two feedback sequences were used (see Methods). One sequence started with positive feedback for the first two trials and had a total of six positive feedbacks in the first ten trials. The other feedback sequence was exactly the opposite. This enabled us to test for path-dependencies by investigating whether the feedback sequence received impacted extent of belief updating. Comparing participants’ starting beliefs $\mu_{0}$ to their ending beliefs, also estimated by the model $\mu_{20}$, revealed that participants who received positive feedback first, shifted their beliefs more in the positive direction from start to end (see S8 Fig). This finding also obtained using model-agnostic starting and ending belief estimates, t(65) = 2.65, p = 0.009. In Model 3, this path-dependency was primarily captured by an increase in the updating bias parameter, $b,$for participants who received positive feedback first, t(65) = 4.48, p < 0.001. However, there was no interaction between feedback order and the relationship between the bias parameter$b$ and anxiety-specific factor scores (interaction, β=0.02, p= 0.81), or indeed with negative affect or depression-specific factor scores (ps>0.54). Although adding both feedback order and anxiety-specific factor scores into the same regression model slightly reduced the strength of the relationship between anxiety scores and the bias parameter from β=-0.14 (p=0.008) to β=-0.10; (p=0.051), these analyses still suggest that feedback order and anxiety have largely independent effects on the bias.

#### Additional models and model comparison

In addition to the four primary models described in the main text, we considered nine supplementary models. Several of these models were not strongly distinguishable from one another within the context of the current task and dataset (see the Model Recoverability subsection below). For completeness, we report them below.

We describe each of the supplementary models in relation to the four primary models presented in the main manuscript: the Bayesian model with biased updating (Model 1), the Bayesian model with unbiased updating (Model 2), the biased Rescorla-Wagner model (Model 3), and the unbiased Rescorla-Wagner model (Model 4).

##### Model 5. RW Model with separate rates of learning following positive versus negative feedback.

This model starts with the unbiased Rescorla-Wagner model (Model 4), but allows the learning rate to differ following positive versus negative feedback. Specifically, the parameter $\eta$ is replaced by two parameters: $\eta_{pos}\in[0,1]$ and $\eta_{neg}\in[0,1]$, as shown below.

Eqn. S1a,b

$\mu_{t}= min(\mu_{t-1} +\eta_{pos}(X_{t}-\mu_{t-1}), 1)$ for $X_{t}=1$

$\mu_{t}= {max(\mu}_{t-1} +\eta_{neg}(X_{t}-\mu_{t-1}),0)$ for $X_{t}=0$

##### Model 6. RW Model with separate biases in learning following positive versus negative feedback.

This model starts with the biased Rescorla-Wagner model (Model 3), but allows for separate biases in learning from positive versus negative feedback. The parameter $b$ is replaced by two parameters: $b_{pos}\in\left[ 0,5 \right], b_{neg}\in[-5,0]$ as shown below.

Eqn. S2a,b

$\mu_{t}= min(\mu_{t-1} +\eta(b_{pos}-u_{t-1}),1)$ for $X_{t}=1$

$\mu_{t}= {max(\mu}_{t-1} +\eta(b_{neg}-\mu_{t-1}), 0)$ for $X_{t}=0$

##### Model 7: Bayesian updating with bias and decay towards unbiased belief

The starting point for this model is model 1 (Bayesian with biased updating). In model 7, the belief distribution decays using the formulations given below towards an unbiased distribution $B(1,1)$ following each feedback-dependent update (for this update see Eqn. 1a,b in the main text).

Eqn. S3a,b

$$\alpha_{t}= {\gamma\alpha}_{t} + \left( 1-\gamma\right)*1$$

$$\beta_{t}= {\gamma\beta}_{t} + \left( 1-\gamma\right)*1$$

The free parameters for this model are $\alpha_{0}\in[0,100]$, $\beta_{0}\in\left[ 0,100 \right], \omega\in[0.1,10]$, and $\gamma\in\left[ 0,1 \right]$. The parameter $\gamma\in[0,1]$ determines the degree to which $\alpha_{t}$ and $\beta_{t}$ persist versus decay from one trial to the next.

##### Model 8: Bayesian updating with bias and decay towards prior belief

In this model, the belief distribution decays (using Eqn. S2a,b) towards the prior distribution estimated for each participant $B(\alpha_{0},\beta_{0})$ after updating each feedback (using Eqn. 1a,b in the main text).

Eqn. S4a,b

$$\alpha_{t}= {\gamma\alpha}_{t} + \left( 1-\gamma\right)\alpha_{0}$$

$$\beta_{t}= {\gamma\beta}_{t} + (1-\gamma)\beta_{0}$$

The free parameters for this model are $\alpha_{0}\in[0,100]$, $\beta_{0}\in\left[ 0,100 \right], \omega\in[0.1,10]$, and $\gamma\in\left[ 0,1 \right]$. The parameter $\gamma\in[0,1]$ determines the size of the decay.

##### Model 9: Bayesian updating: separate reporting and updating distributions

This model divides the belief distribution that participants used for both reporting beliefs and updating beliefs into two separate Beta distributions, one for reporting and one for updating. The rationale for this separation is to capture behavior such as the following: a participant may update his beliefs substantially to new information (i.e., have a wide updating distribution), but he may report beliefs close to the mean of the distribution (i.e., have a narrow reporting distribution).

The reporting distribution is still denoted by $\hat{u} \sim B(\alpha_{t}, \beta_{t}$). The new distribution for updating beliefs is now denoted by $B(\alpha_{t}^{u}, \beta_{t}^{u})$, where the superscript *u* denotes ‘updating’ and is used to differentiate these parameters from the $\alpha_{t}$ and $\beta_{t}$ of the reporting distribution. The parameters of the updating beliefs distribution are updated according to Eqn. S5ab, which replaces Eqn. 1a,b in the main text.

Eqn. S5a,b

$$\alpha_{t}^{u}= \alpha_{t-1}^{u}+X_{t}$$

$$\beta_{t}^{u}=\beta_{t-1}^{u} +(1-X_{t})$$

The $\alpha_{t}$ and $\beta_{t}$ from the reporting distribution are then calculated directly from the updating belief distribution parameters (without being progressively updated themselves). This is described below (Eqns S6 and S7a,b). The free parameters for this model are $\alpha_{0}^{u}\in[0,100]$, $\beta_{0}^{u}\in\left[ 0,100 \right],$and $v\in[1,1000]$. The precision parameter ($\nu$) corresponds to the width of the reporting distribution.

Eqn. S6

$$\mu_{t}= \frac{\alpha_{t}^{u}}{\alpha_{t}^{u}+\beta_{t}^{u}}$$

Eqn. S7a,b

$$\alpha_{t}= v*\mu_{t}$$

$$\beta_{t}= v-\alpha_{t}$$

##### Model 10: Bayesian updating: separate reporting and updating distributions with bias

This model is the same as model 9 except that it allows for biased updating, i.e. includes a bias parameter $\omega.$ Eqn. S5a,b are replaced with Eqn. S8a,b below. The free parameters for this model are $\alpha_{0}^{u}\in[0,100]$, $\beta_{0}^{u}\in[0,100]$, $\omega\in[0.1,10]$, $v\in[1,1000]$.

Eqn. S8a,b

$$\alpha_{t}^{u}= \alpha_{t-1}^{u}+{\omega X}_{t}$$

$$\beta_{t}^{u}=\beta_{t-1}^{u} +\frac{1}{\omega} (1-X_{t})$$

##### Model 11: Bayesian updating: separate reporting and updating distributions with bias and decay towards neutral.

This model takes model 10 and introduces the decay term from model 7. The updating distribution parameters $\alpha^{u}, \beta^{u}$ decay back to a neutral distribution centered around 50% using Eqn S3a,b. The free parameters for this model are $\alpha_{0}^{u}\in[0,100]$, $\beta_{0}^{u}\in[0,100]$, $\omega\in[0.1,10]$, $\gamma\in[0.2,1]$, $v\in[1,1000]$.

##### Model 12: Bayesian updating: separate reporting and updating distributions with bias and decay towards prior belief.

This model takes model 10 and introduces the decay term from model 8. The updating distribution parameters $\alpha^{u}, \beta^{u}$ decay back to their starting values using Eqn S4a,b. The free parameters for this model are $\alpha_{0}^{u}\in[0,100]$, $\beta_{0}^{u}\in[0,100]$, $\omega\in[0.1,10]$, $\gamma\in[0.2,1]$, $v\in[1,1000]$.

##### Model 13: Bayesian updating: separate reporting and updating distributions with positive and negative updating biases

This model is the same as model 10 except that it has two bias parameters, one for positive and one for negative feedback. This allows us to model individual differences that are specific to updating from positive feedback or from negative feedback as opposed to simply assessing the difference between the two. The free parameters for this model are $\alpha_{0}^{u}\in[0,100]$, $\beta_{0}^{u}\in[0,100]$, $\omega_{pos}\in[0.1,10]$,$\omega_{neg}\in\left[ 0.1,10 \right], v\in[1,1000]$.

#### Model Recoverability

To determine whether the different models were distinguishable from one another in the context of our task dataset, we simulated fake data from each possible model and performed model comparison using all thirteen models. A model was considered as distinguishable (i.e., identifiable) if it was chosen as the winning model a large percentage of the time when it was indeed the true (generative) model.

Fake data for 100 participants was generated for each of the 13 models. The confusion matrix in S4 Fig shows, for each model, the percentage of fake participants where each model was chosen as the best fit, based on a comparison of BIC values across models for that one simulated participant’s dataset. A large value on the diagonal indicates that the true (generative) model was identified as the winning model most of the time. It can be seen that models 1-4 (reported in the main text) have good model identifiability. This is also true for the two supplementary RW models 5 and 6. However, as discussed in the next section, models 5 and 6 provided a worse fit to participants data than our winning model, model 3.

#### Model Comparison and robustness of correlational results to model selection.

Self belief data: The biased RW model (Model 3; reported in the main manuscript) was estimated to have an exceedance probability of 0.67 when tested against the additional alternate models detailed in this supplemental section. The second-best model was model 10, which was a Bayesian model with biased updating and separate reporting and updating distributions. This model, however, had a much lower exceedance probability of 0.25, as well as lower model recoverability in the previous analysis. Nevertheless, this second-best model showed similar results as the best model (Model 3) with regards to anxiety and depression. In Model 10, the updating bias parameter $\omega$ was significantly correlated with the anxiety factor (r(64)=-0.31, uncorrected p=0.012), and the prior mean $\mu_{0}$ (i.e. $\frac{\alpha_{0}^{u}}{\alpha_{0}^{u}+\beta_{0}^{u}}$) was significantly correlated with the depression factor (r(64)=-0.34, uncorrected p=0.005). The third-best model (Model 5), with an exceedance probability of 0.07, was a Rescorla-Wagner model which estimated separate learning rates for positive and negative feedback. This model also showed similar results with respect to anxiety and depression; the prior mean $\mu_{0}$ significantly correlated with the depression factor (r(64)=-0.34, uncorrected p=0.005) and updating bias (parameterized as the ratio of positive and negative learning rates $\frac{\eta_{pos}}{\eta_{neg}}$) significantly correlated with the anxiety factor (r(64)=-0.34, uncorrected p=0.005); note that the depression and anxiety correlations are coincidently the same up to three decimal places.

Other belief data: The biased RW model (Model 3; reported in the main manuscript) was estimated to have an exceedance probability of 0.62 when tested against the additional alternate models detailed in this supplemental section. This is very close to that reported above for self belief data. For the second-best model (Model 5; exceedance probability of 0.33) and third-best model (Model 12; exceedance probability of 0.05), scores on the depression-specific factor showed a similar non-significant trend towards a negative correlation with participants’ initial other-referent belief (r(64)=-0.23; p=0.058; r(64)=-0.22; p=0.076, respectively) to that observed for model 3 (r(64)=-0.23; p = 0.066). As for model 3, when model 5 or model 12 was implemented, there was no significant relationship between scores on the anxiety-specific factor and updating bias for other-referent beliefs, ps>.5.

#### Incorporating information about profile strength: a supplementary set of 4 models with model 3 taken as the starting point.

Models 1-13 treat the feedback that a participant received as a binary quantity – the participant was either chosen or not. However, it is also possible that information about the relative strength of the profiles shown on each trial led participants to form expectations about whether they would be chosen or not and to experience a varying degree of surprise based on this expectation and the feedback provided. This in turn might have influenced the extent to which they updated their beliefs. To explore this possibility, we constructed and tested four additional models. We used model #3, the best model from the primary model comparison analysis, as the base model for this new set of models and compared the fit of these news models directly against that of model #3.

##### Model 3a: RW Model with a continuous measure of outcome surprise

The first additional model incorporates an estimate of outcome surprise using the difference in profile strength for the candidate versus the other participant whose profile was presented on the trial in question. Profile popularity (see Results) is used as an index of profile strength; this is defined as the number of times that the profile was selected relative to that of other participants in session 2. Surprise is defined as the difference in profile popularity normalized between 0-1 across all potential pairings of profiles: a 1 indicates that least popular profile was selected over the most popular, and 0 indicates the opposite outcome.

Surprise $S_{t}$ is incorporated into model 3 through the modulation of learning rate $\eta_{t}$ given by equation S9. The learning rate is then used in equation 1 exactly as before (reproduced for convenience below):

Eqn. 1

$\mu_{t}= {min(\mu}_{t-1} +\eta\left( {bX}_{t}-\mu_{t-1} \right),1)$

Eqn. S9

$\eta_{t}= \eta\left( 1 +\phi\left( S_{t}-0.5 \right) \right)$

If $\phi$ is 0, this model reduces to model #3 exactly. For $\phi$>0 (up to a maximum of 2), the magnitude of belief updates is correlated with the magnitude of surprise. A candidate would increase his/her belief of being in the most popular half *more* after receiving feedback that he/she had been chosen over a better profile, and *less* after receiving feedback that he/she had been chosen over a worse profile. And for negative feedback, the candidate would decrease his/her beliefs *more* if not chosen over a worse profile and *less* if not chosen over a better profile.

We compared this new *continuous surprise* model (#3a) to model #3 using BIC. The average BIC across participants favored model #3 (model #3a: mean BIC = -74.11; model #3: mean BIC = -75.63; t = -4.60 p < 0.001; paired t-test), indicating that the added flexibility of this new model was not warranted. The difference in model fit also did not correlate with scores on any of the three latent factors of Internalizing symptoms (ps>.2). The estimates for the other parameters in model #3a were very similar to those for model #3; and both the correlations between prior belief and depression scores (p=0.005) and between bias and anxiety scores (p=0.015) remained significant.

##### Model 3b: RW Model with a binary indicator of outcome surprise

The second additional model uses a binarized form of surprise (i.e., $S_{t}$ is either 0 or 1) but is otherwise identical to the *continuous surprise* model. Participants positively update their belief of being in the most popular half more if they were chosen over a stronger profile and less if they were chosen over a weaker profile and the reverse if they were not chosen (updating more if not chosen over a weaker profile and less if not chosen over a stronger profile), but they do not use graded relative profile popularity.

This *binary surprise* model (#3b) also did not outperform model #3, model #3b: mean BIC = -74.75; model #3: mean BIC = -75.63; t = -1.77 p = 0.09). The two surprise models did not perform significantly differently (t=1.39; p=0.17).

##### Model 3c: RW Model with two types of independent feedback

In a third additional model, outcome feedback (i.e., whether or not the participant was selected) and binary relative profile popularity influence belief updating independently. In this model, the feedback $X_{t}$ rather than the learning rate is modified. The new feedback $Z_{t}$ (replacing $X_{t}$ in equation 1) is given by:

Eqn. S10

$Z_{t}=\lambda X_{t}+{(1-\lambda)Y}_{t}$

Here, $Y_{t}$ is an indicator of profile superiority, where $Y_{t}$=1 if the profile popularity of the participant is greater than that of the other participant shown on trial *t*, and $Y_{t}$=0 if the profile popularity of the participant is less than that of the other participant. The parameter $\lambda$ is a mixture weight (between 0 and 1), which determines how much the participant uses one type of feedback over the other for belief updating.

The average BIC again favored model #3 (*combined feedback* model #3c: mean BIC = -74.14; model #3 mean BIC = -75.63; t = -4.15 p < 0.001), suggesting that allowing profile superiority to independently influence belief updating does not improve model fit.

##### Model 3d: RW Model with only profile superiority as feedback

Setting $\lambda$=0, corresponds to a model in which only profile superiority is used to update beliefs and not explicit feedback as to whether the participant’s profile was chosen or not. This *profile superiority* model (#3d) performed much more poorly than model #3 (model #3d: mean BIC = -64.99; model #3 =-75.63, t=-7.26, p<0.001.

##### Summary of model comparison for the additional models #3a-d against model #3

Overall, these results indicate that model fit is not improved by explicitly modeling a surprise signal that depends on the relative strength of the profiles in each pair. This is perhaps expected given that (i) participants were given true feedback, so it would be relatively rare that a participant was shown a much less popular profile that was chosen over their profile and (ii) participants showed insensitivity to their profile popularity as demonstrated by the lack of a correlation between prior beliefs and profile popularity (see Results).
