# Supplementary material for: Biased belief priors versus biased belief updating: Differential correlates of depression and anxiety
Source: PLoS Comput Biol. 2022 Aug 15;18(8):e1010176. doi: 10.1371/journal.pcbi.1010176 (PMC9377597; doi:10.1371/journal.pcbi.1010176)
Supplement: S1 Table — Summary scores for each of the questionnaires administered are shown for participants in the current study as well as for those from a prior study [25], which included individuals with MDD, GAD, healthy controls and an unselected community sample (columns 2 to 5, respectively). In addition to giving the mean and SD for scores on each measure for each group, we also report the number of our current participants who fell within one standard deviation of the mean for the MDD and GAD groups, see square brackets and note 2. STAI = Spielberger State-Trait Anxiety Inventory (form Y; [23]); MASQ-AD/MASQ-AA = anhedonic depression and anxious arousal subscales for the Mood and Anxiety Symptoms Questionnaire [21,22]; PSWQ = Penn State Worry Questionnaire [20]; CESD = Center for Epidemiologic Studies Depression Scale [24]. (DOCX) [file pcbi.1010176.s003.docx]

|  | **Current Study**  **n=66** | **Major Depressive Disorder (MDD)**  **n=20**  (Gagne et al. 2020) | **Generalized Anxiety Disorder (GAD)**  **n=12**  (Gagne et al. 2020) | **Healthy Controls**  **n=24**  (Gagne et al. 2020) | **Unselected Community Sample**  **n=30**  (Gagne et al. 2020) |
| --- | --- | --- | --- | --- | --- |
| **STAI^1^** mean ± sd | 42.2 ± 10.0  [8,15]^2^ | 59 ± 6 | 58 ± 9 | 40 ± 12 | 36 ± 12 |
| **MASQ-AD** mean ± sd | 55.7 ± 14.4  [10, 27] ^2^ | 80 ± 10 | 74 ± 16 | 55 ± 18 | 50 ± 20 |
| **MASQ-AA** mean ± sd | 23.8 ± 7.4  [33, 35] ^2^ | 28 ± 7 | 33 ± 10 | 21 ± 4 | 22 ± 6 |
| **PSWQ** mean ± sd | 53.3 ± 13.1  [45, 8] ^2^ | 62 ± 14 | 76 ± 9 | 52 ± 13 | 42 ± 15 |
| **CESD^1^** mean ± sd | 13.6 ± 7.31  [10,20] ^2^ | 30 ± 9 | 30 ± 14 | 12 ± 8 | 10 ± 11 |

^1^averaged across session 2 and 3

^2^number of current participants within 1SD of mean of MDD and GAD patient groups respectively
