# Supplementary material for: Biased belief priors versus biased belief updating: Differential correlates of depression and anxiety
Source: PLoS Comput Biol. 2022 Aug 15;18(8):e1010176. doi: 10.1371/journal.pcbi.1010176 (PMC9377597; doi:10.1371/journal.pcbi.1010176)
Supplement: S2 Table — Participants’ standardized scores for the general factor, anxiety-specific factor and depression-specific factor (columns) are correlated against model agnostic measures of prior beliefs and belief updating (rows). Participants reported believes about themselves (‘self’; first three rows) and for another randomly chosen participant (‘other’; last three rows). Pearson correlations were used to examine the relationship between scores on the three latent symptom factors and our two model agnostic indices of interest. Here uncorrected p values are given, p values that survive multiple comparison correction at p < .05 are indexed by *. (DOCX) [file pcbi.1010176.s004.docx]

|  | **General Factor** | **Depression-Specific Factor** | **Anxiety-Specific Factor** |
| --- | --- | --- | --- |
| **Starting Belief (self)** | r(64) = 0.09, p = 0.485 | r(64) = -0.36, p = 0.003* | r(64) = -0.05, p = 0.684 |
| **Ending – Starting Belief (self)** | r(64) = 0.04, p = 0.766 | r(64) = 0.13, p = 0.28 | r(64) = -0.23, p = 0.065 |
| **Ending Belief (self)** | r(64) = 0.12, p = 0.326 | r(64) = -0.26, p = 0.034 | r(64) = -0.24, p = 0.049 |
| **Starting Belief (other)** | r(64) = -0.08, p = 0.531 | r(64) = -0.14, p = 0.25 | r(64) = 0.06, p = 0.619 |
| **Ending – Starting Belief (other)** | r(64) = -0.11, p = 0.378 | r(64) = 0.06, p = 0.648 | r(64) = -0.09, p = 0.486 |
| **Ending Belief (other)** | r(64) = -0.15, p = 0.226 | r(64) = -0.06, p = 0.605 | r(64) = -0.02, p = 0.857 |
