# Supplementary material for: Biased belief priors versus biased belief updating: Differential correlates of depression and anxiety
Source: PLoS Comput Biol. 2022 Aug 15;18(8):e1010176. doi: 10.1371/journal.pcbi.1010176 (PMC9377597; doi:10.1371/journal.pcbi.1010176)
Supplement: S3 Table — Each regression analysis uses scores on one of the three latent factor dimensions as the dependent variable and parameter estimates for both μ0 and b as predictors. Parameter estimates were obtained using the winning model: Model 3 the “biased RW” model. These regression analyses confirmed our original finding of a significant relationship between μ0 and depression-specific affect and a significant relationship between b and anxiety-specific affect for self-referential judgements. (DOCX) [file pcbi.1010176.s005.docx]

| ***Depression-specific model*** | **Coefficient** | **Std. Error** | **t-statistic** | **P-value** |
| --- | --- | --- | --- | --- |
| **intercept** | 0.8002 | 0.349 | 2.295 | 0.025 |
| **updating bias (**$\boldsymbol{b}$**)** | 0.0993 | 0.307 | 0.323 | 0.747 |
| **prior belief (**$\boldsymbol{\mu}_{\mathbf{0}}$**)** | -1.8053 | 0.696 | -2.594 | 0.012 |
| ***Anxiety-specific model*** | **Coefficient** | **Std. Error** | **t-statistic** | **P-value** |
| **intercept** | 0.5462 | 0.348 | 1.570 | 0.122 |
| **updating bias (**$\boldsymbol{b}$**)** | -0.8648 | 0.306 | -2.824 | 0.006 |
| **prior belief (**$\boldsymbol{\mu}_{\mathbf{0}}$**)** | 0.6445 | 0.695 | 0.928 | 0.357 |
| ***General negative affect model*** | **Coefficient** | **Std. Error** | **t-statistic** | **P-value** |
| **intercept** | -0.2589 | 0.367 | -0.706 | 0.483 |
| **updating bias (**$\boldsymbol{b}$**)** | 0.3213 | 0.323 | 0.995 | 0.324 |
| **prior belief (**$\boldsymbol{\mu}_{\mathbf{0}}$**)** | -0.1272 | 0.732 | -0.174 | 0.863 |
