# Supplementary material for: Biased belief priors versus biased belief updating: Differential correlates of depression and anxiety
Source: PLoS Comput Biol. 2022 Aug 15;18(8):e1010176. doi: 10.1371/journal.pcbi.1010176 (PMC9377597; doi:10.1371/journal.pcbi.1010176)
Supplement: S4 Table — Each regression analysis uses scores on one of the three latent factor dimensions as the dependent variable and parameter estimates for both μ0 and b as predictors. Parameter estimates were obtained using the winning model: Model 3 the “biased RW” model. These additional regressions confirmed the findings from our correlational analyses, with neither μ0 or b significantly predicting scores on any of the three latent dimensions, and with only a trend-level relationship between other-belief μ0 and depression-specific affect, (t = -1.870, p = 0.066). (DOCX) [file pcbi.1010176.s006.docx]

| ***Depression-specific model*** | **Coefficient** | **Std. Error** | **t-statistic** | **P-value** |
| --- | --- | --- | --- | --- |
| **intercept** | 0.9018 | 0.528 | 1.709 | 0.092 |
| **updating bias (**$\boldsymbol{b}$**)** | 0.1138 | 0.267 | 0.426 | 0.671 |
| **prior belief (**$\boldsymbol{\mu}_{\mathbf{0}}$**)** | -1.8787 | 1.004 | -1.870 | 0.066 |
| ***Anxiety-specific model*** | **Coefficient** | **Std. Error** | **t-statistic** | **P-value** |
| **intercept** | -0.1466 | 0.540 | -0.272 | 0.787 |
| **updating bias (**$\boldsymbol{b}$**)** | -0.2004 | 0.273 | -0.734 | 0.466 |
| **prior belief (**$\boldsymbol{\mu}_{\mathbf{0}}$**)** | 0.6910 | 1.027 | 0.673 | 0.504 |
| ***General negative affect model*** | **Coefficient** | **Std. Error** | **t-statistic** | **P-value** |
| **intercept** | 0.3078 | 0.541 | 0.569 | 0.572 |
| **updating bias (**$\boldsymbol{b}$**)** | -0.0428 | 0.274 | -0.156 | 0.876 |
| **prior belief (**$\boldsymbol{\mu}_{\mathbf{0}}$**)** | -0.4680 | 1.030 | -0.454 | 0.651 |
