# Supplementary figures and images for: Biased belief priors versus biased belief updating: Differential correlates of depression and anxiety
Source: PLoS Comput Biol. 2022 Aug 15;18(8):e1010176. doi: 10.1371/journal.pcbi.1010176 (PMC9377597; doi:10.1371/journal.pcbi.1010176)

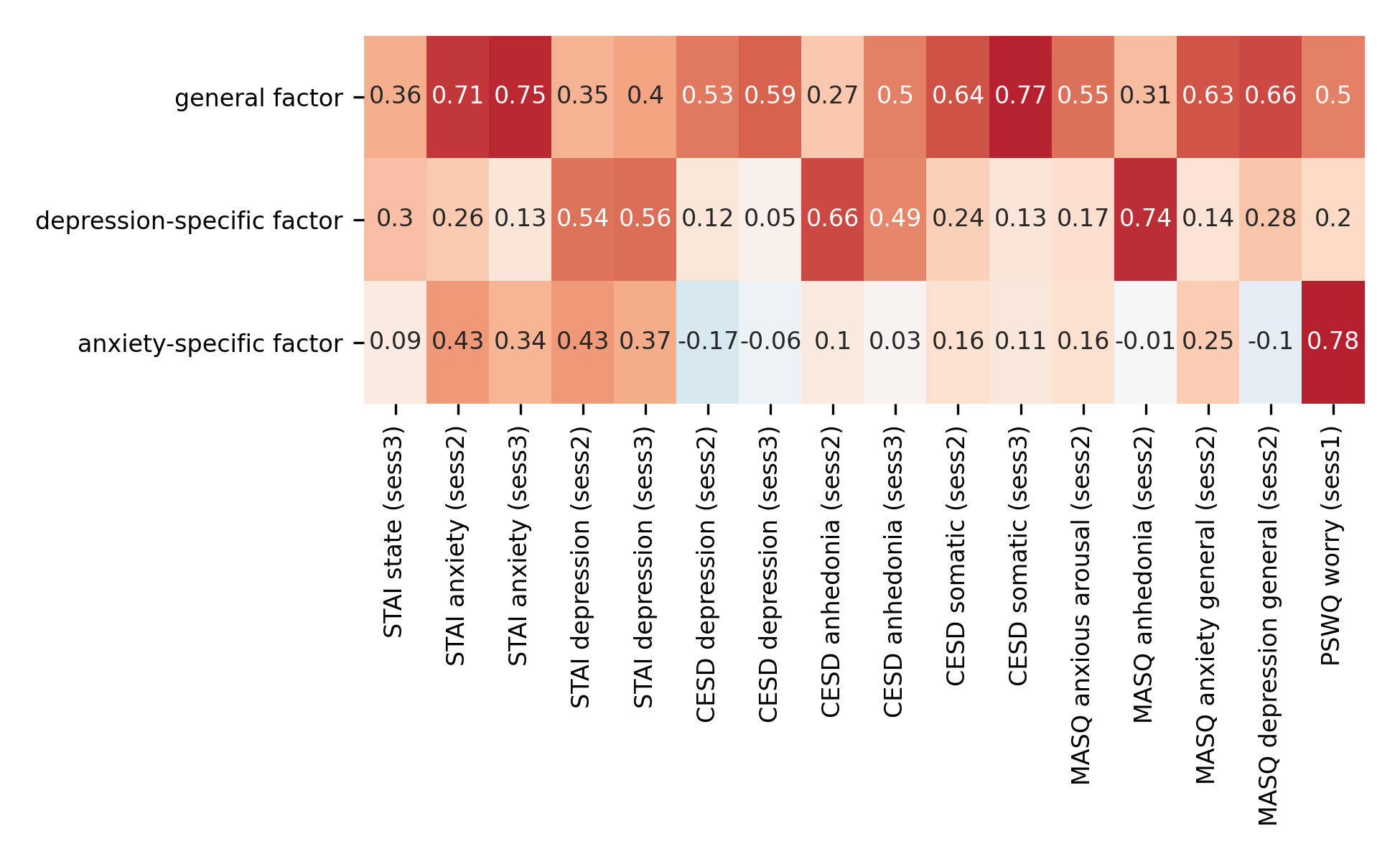

Supplement: S1 Fig — Factor loadings from a previously published bifactor analysis of item-level responses to self-report questionnaire measures of anxiety and depression [25] was used to estimate current participants’ scores on three orthogonal latent factors: a general Negative Affect factor and Anxiety and Depression specific factors. To check the concurrent validity of these factors in the current sample, we correlated scores on each factor (y axis) with scores on each of the subscales administered (x axis). Scores on the general factor showed a positive correlation with scores across all of the subscales. Scores on the depression-specific factor correlated most strongly with scores on the anhedonia-related subscales (e.g., MASQ anhedonia; CESD anhedonia). Scores on the anxiety-specific factor correlated most strongly with scores on the PSWQ. Self-report questionnaire measures were administered at the end of one or more sessions; for details, see Methods. (TIF) [file pcbi.1010176.s007.tif]

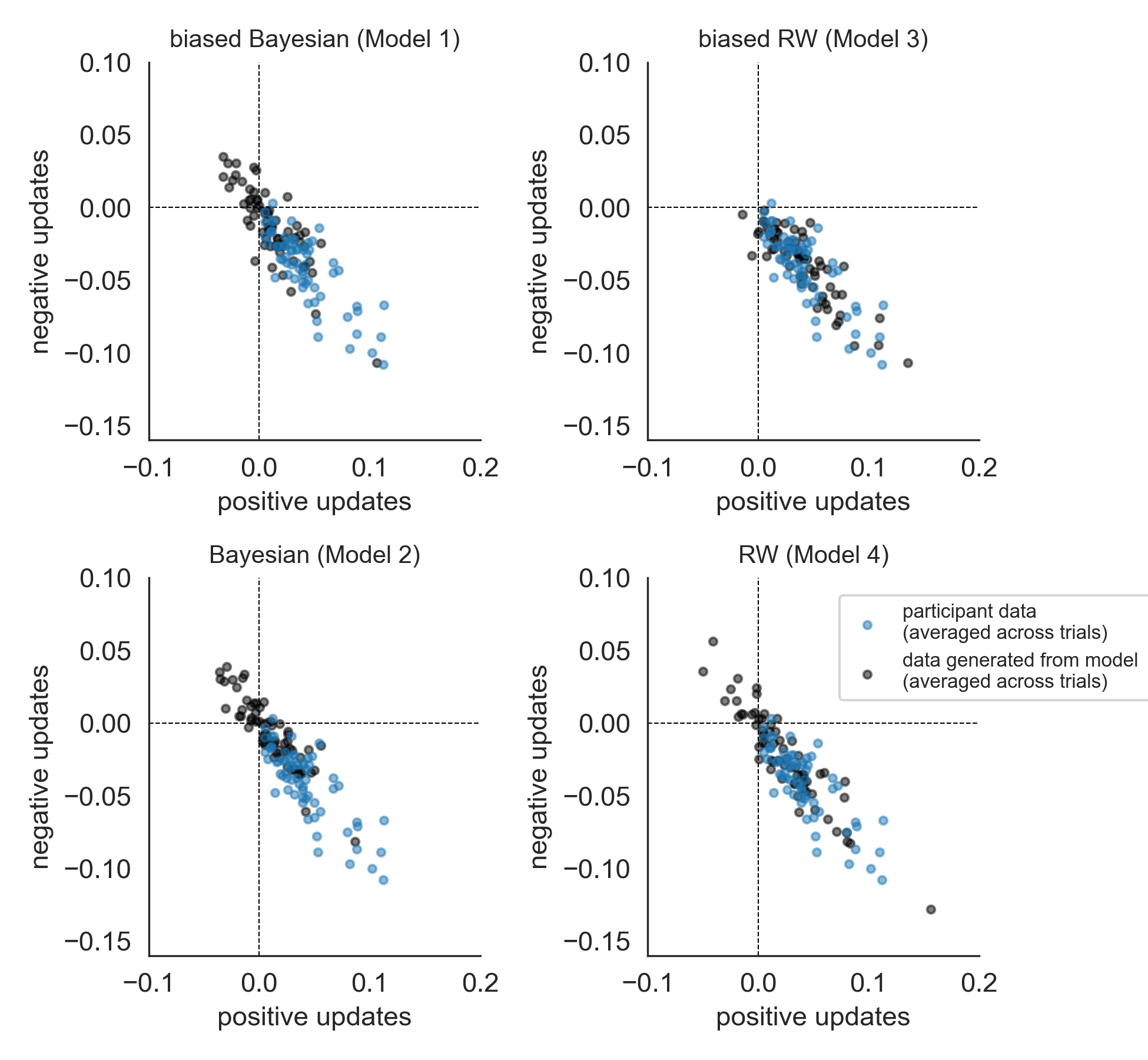

Supplement: S2 Fig — The four models considered were each used to simulate 20 new estimated judgements for each participant for each trial, using that participant’s parameter estimates for the model in question. Here, we plot the trial-to-trial changes in judgments, averaged separately for trials following positive (x axis) versus negative (y axis) feedback, for both the simulated data (in black) and the original data (in blue). Each panel shows the results for a separate model. These models differ along two dimensions: (i) whether beliefs are updated either according to Bayesian or Rescorla-Wagner (RW) principles (separated by column), and (ii) whether a bias in updating after negative versus positive feedback was incorporated (separated by row). It can be seen that simulated data from Model 3, ‘the biased RW model’ (top right panel) provides a better match to the distribution of participants’ updates than is the case for the other three models considered; a Kolmogorov-Smirnov test confirms that there are significant differences between the simulated and real data distributions for models 1, 2 and 4 (p<0.001) but not for model 3 (p = 0.5). Note, positive feedback should lead to a positively signed update and negative feedback to a negatively signed update, hence data points in all but the bottom right quadrant reflect updating in the opposite direction to the feedback provided. Note further that beliefs in the model are sampled, and so need not normatively occupy that quadrant. (TIF) [file pcbi.1010176.s008.tif]

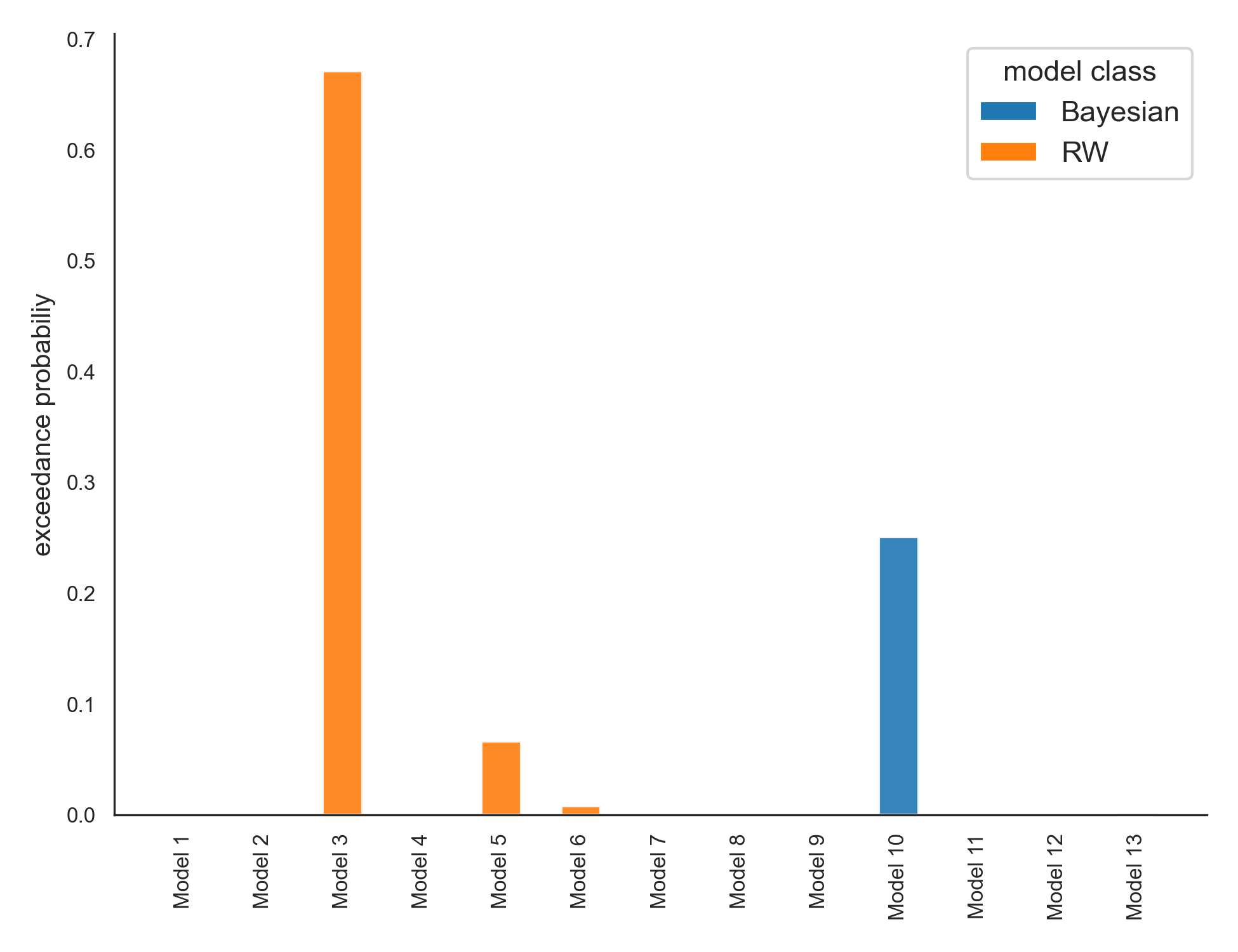

Supplement: S3 Fig — Models were compared using exceedance probability, which is defined as the probability that a model is the most prevalent at the population level. Exceedance probabilities are estimated using hierarchical Bayesian inference [29]. The biased RW model (Model 3) was estimated to be the most prevalent (most likely) model, with an exceedance probability of 0.67. The second most prevalent model was Model 10, a biased Bayesian model with separate reporting and updating distributions. This model had a much lower exceedance probability of 0.25. For descriptions of each model, see S2 Text. (TIF) [file pcbi.1010176.s009.tif]

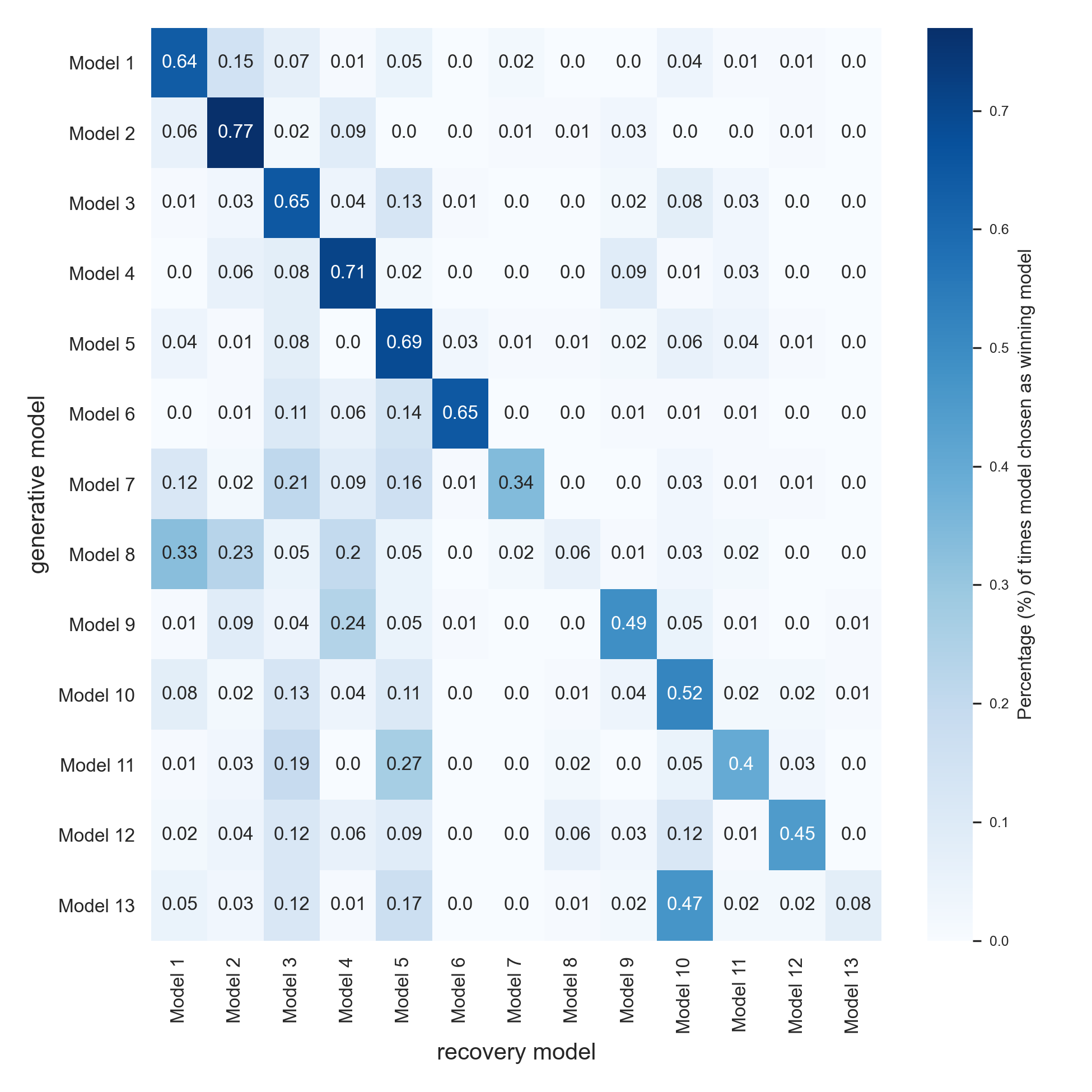

Supplement: S4 Fig — Each of the models on the y-axis were considered, in turn, to be the true model and used to generate data for 100 new participants; new parameter values were chosen for each simulated participant by sampling from distributions informed by the distribution of actual parameter estimates. The simulated data were fit by each of the possible models listed on the x-axis and the percentage of participants for which each ‘recovery’ model had the best penalized fit (BIC) is shown in the cells along each row. The percentages add up to 100% for each simulated dataset (i.e., across each row). This was completed for all the generative models on the y-axis. A high percentage along the diagonal indicates high model recoverability—i.e., that the model that generated the data was chosen as the best fitting model against all others. Models 1 to 4 correspond to the four main models presented in the main manuscript. Models 5–13 correspond are presented in S2 Text, Supplemental Model-based Analyses: Additional models and model comparison. (TIF) [file pcbi.1010176.s010.tif]

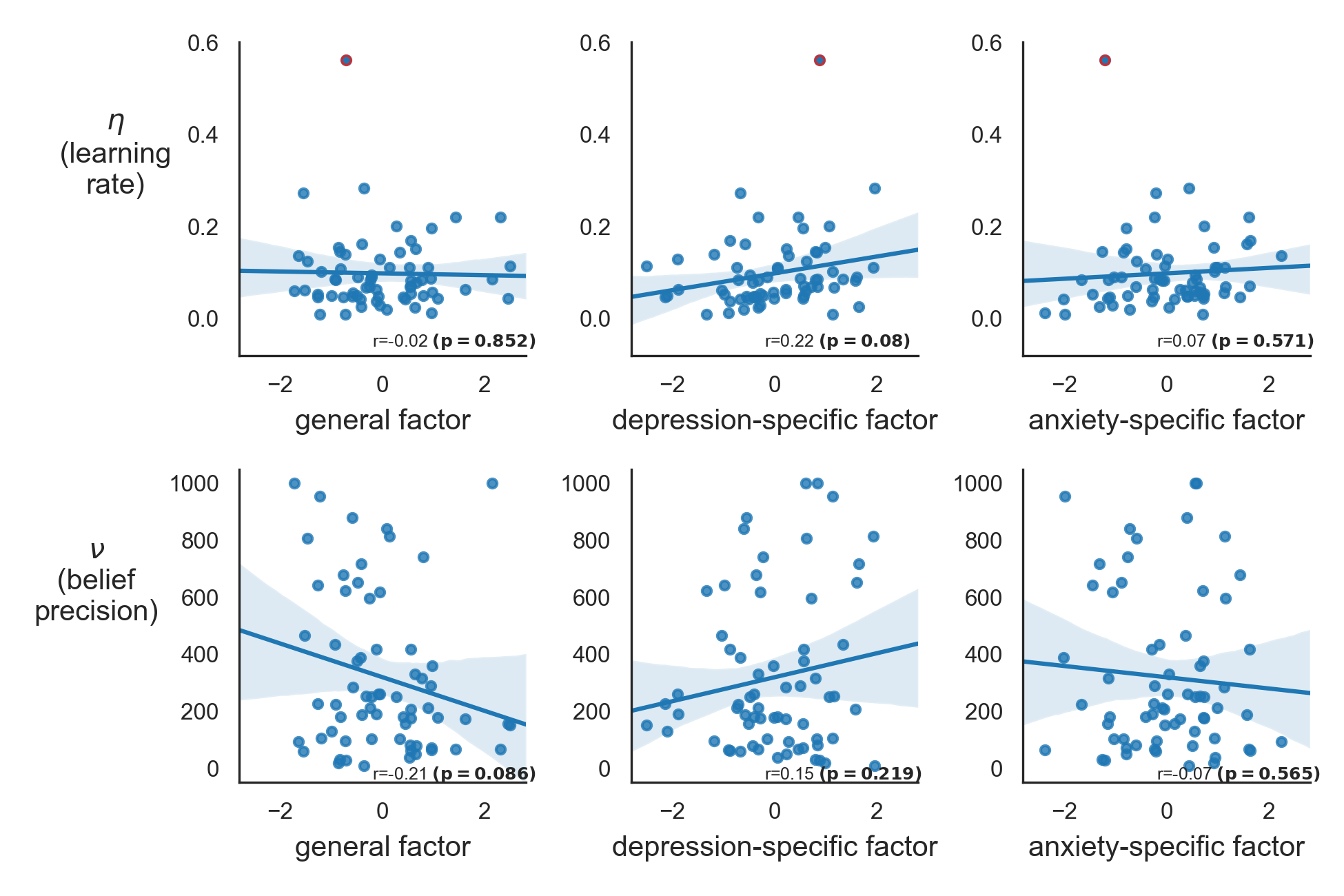

Supplement: S5 Fig — Participants’ standardized scores for the general factor, anxiety-specific factor and depression-specific factor (x axes) are plotted against the two other parameters in the main model (Model 3: biased RW). η is the estimated learning rate, which measures the absolute amount that beliefs are updated in response to feedback, and ν is the estimated precision of the reporting belief distribution, which measures how close participants’ reported beliefs are to the mean of their belief distribution. Neither η nor ν significantly correlated with scores on any of the three Internalizing symptom factors (uncorrected p-values shown here). After removing the outlier for learning rate (outlined in red), the correlation between η and scores on the anxiety-specific factor rose to r(64) = 0.25 (uncorrected p = 0.048), but this does not survive correction for multiple comparisons (corrected p = 0.19). (TIF) [file pcbi.1010176.s011.tif]

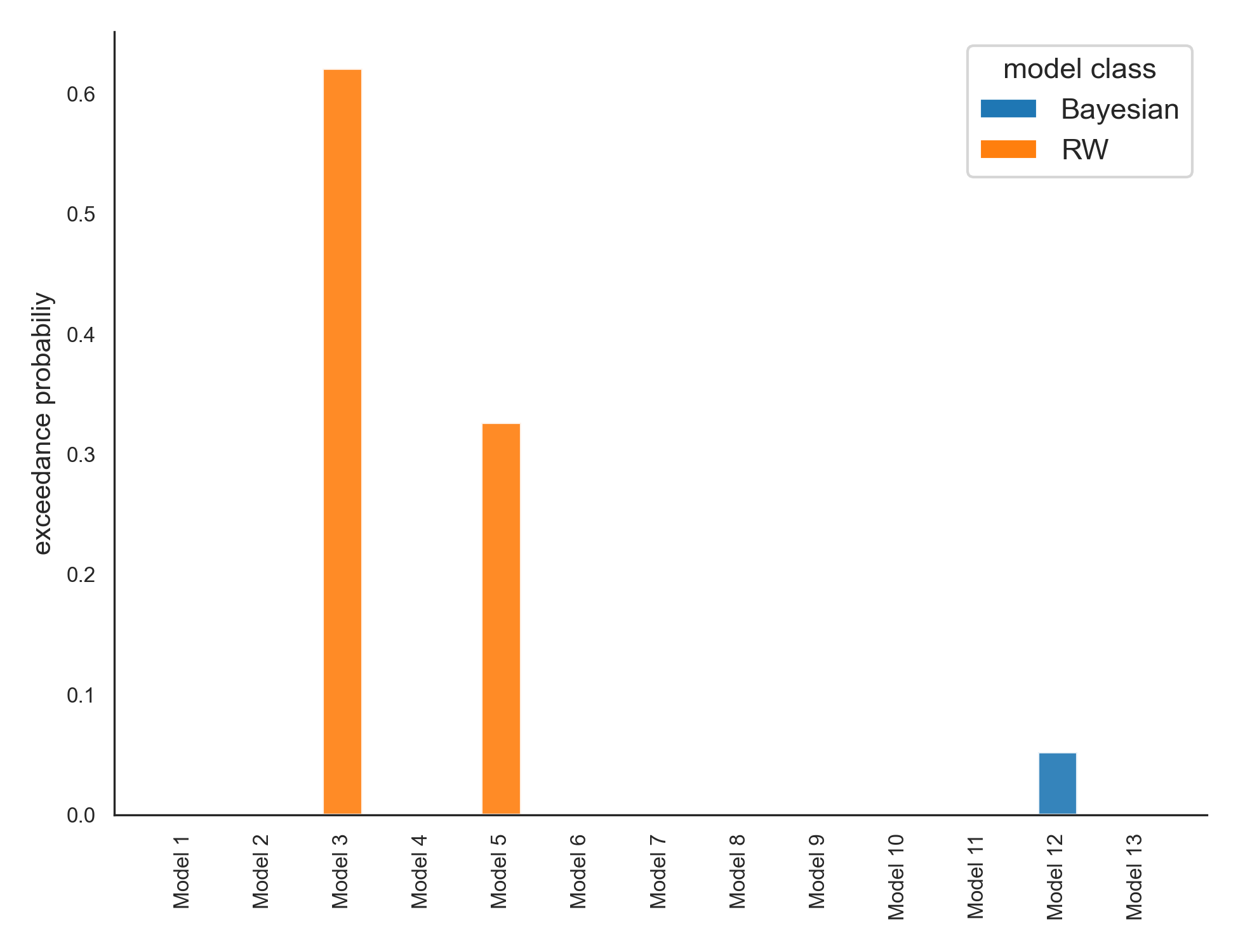

Supplement: S6 Fig — Participants were also asked to report and updating beliefs about another randomly chosen participant. The same thirteen models were fit to these other-referent belief data and compared using exceedance probability. The biased RW model (Model 3) was again estimated to be the most prevalent (most likely) model, with an exceedance probability of 0.62. This model comparison result closely match that for the self-referent data (S3 Fig). (TIF) [file pcbi.1010176.s012.tif]

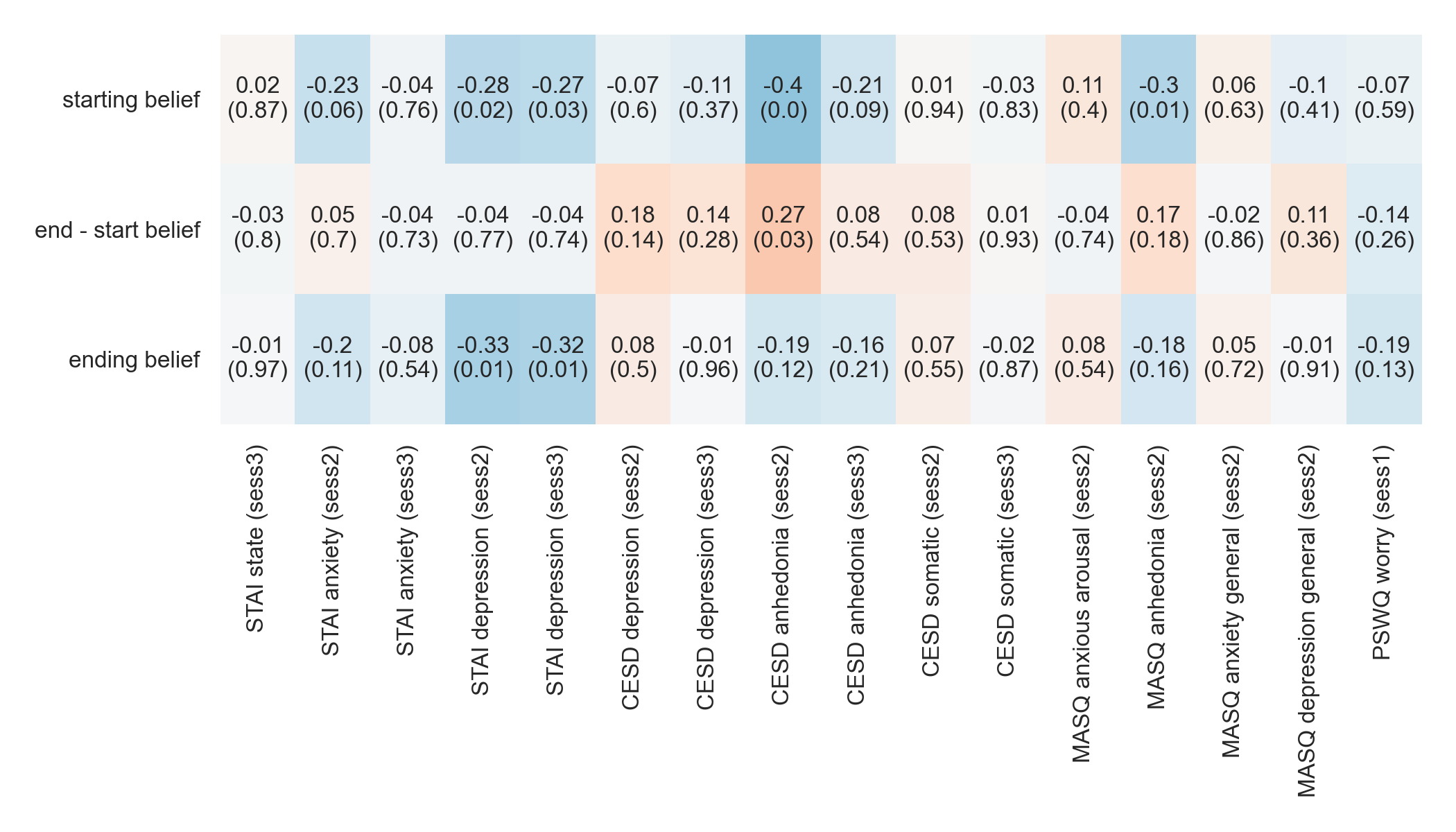

Supplement: S7 Fig — P-values are shown in parentheses underneath Pearson correlation values. In line with the factor score results, subscales tapping anhedonic depression were negatively correlated with starting belief (CESD anhedonia, MASQ anhedonia, STAI depression). These model-agnostic measures of belief updating and broad-brush measures of anxiety and depressive symptomatology, that do not dissociate variance common to both anxiety and depression from that unique to anxiety or depression, do not provide a clear picture of a relationship between negative affect and bias in belief-updating. (TIF) [file pcbi.1010176.s013.tif]

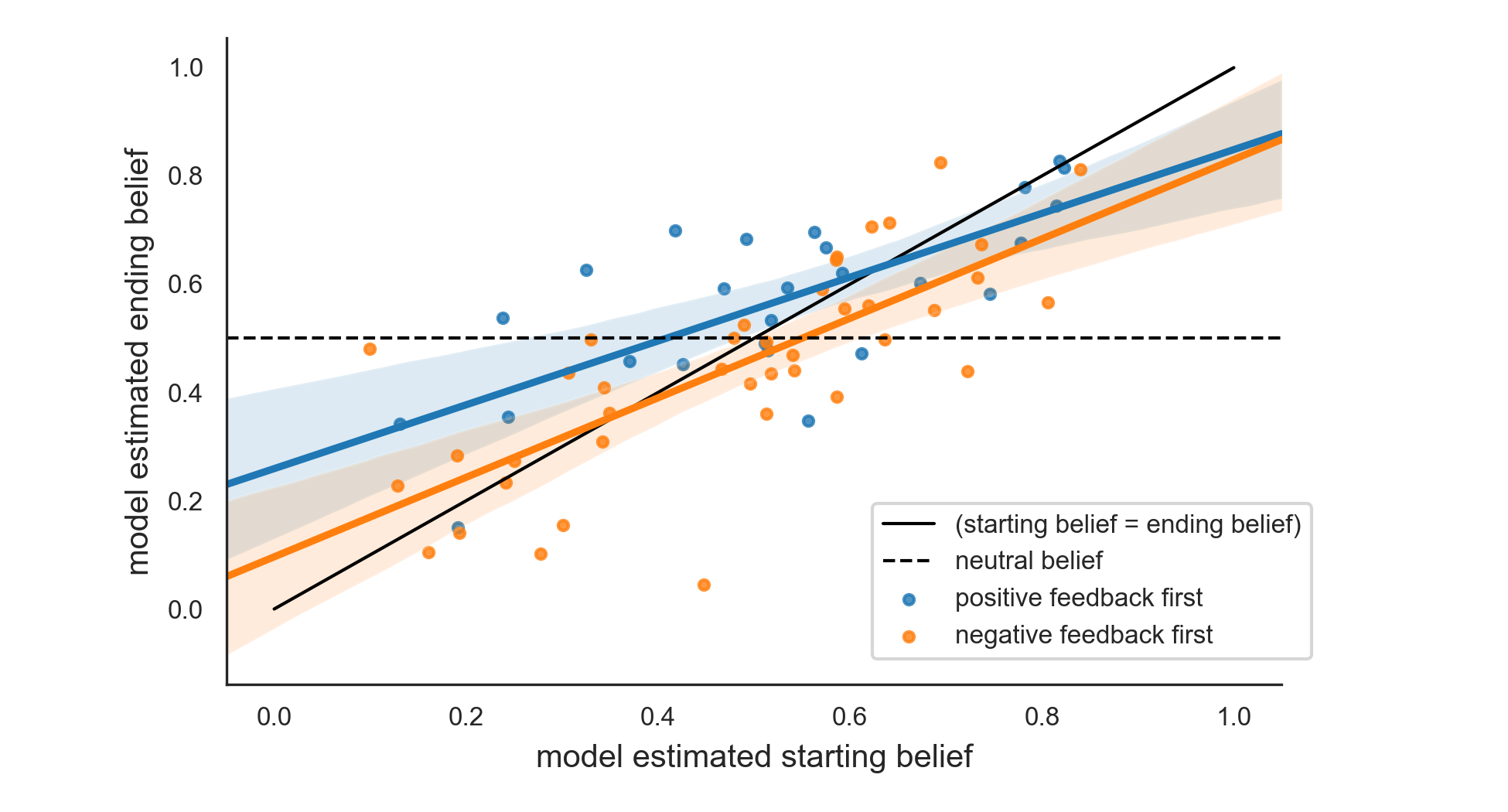

Supplement: S8 Fig — Participants beliefs, as estimated by the model, are plotted for the start and for the end of the feedback period. Participants are split into two groups based on which feedback sequence they received: the positive-first feedback sequence, which started with positive feedback for the first two trials and had a total of six positive feedbacks in the first ten trials, or the negative-first feedback sequence, which was exactly the opposite. In both groups, participants update towards a neutral belief (i.e., 50%) after receiving twenty instances of balanced feedback. This partial updating towards 50% is indicated by the two regression lines, both which have a slope (0.49 for positive-first; 0.74 for negative-first) significantly greater than 0 and significantly less than 1. However, participants who received positive feedback first shifted their beliefs more in the positive direction from start to end (independent t-test, difference between groups for ending-starting beliefs, t(65) = 4.48, p < 0.001). (TIF) [file pcbi.1010176.s014.tif]

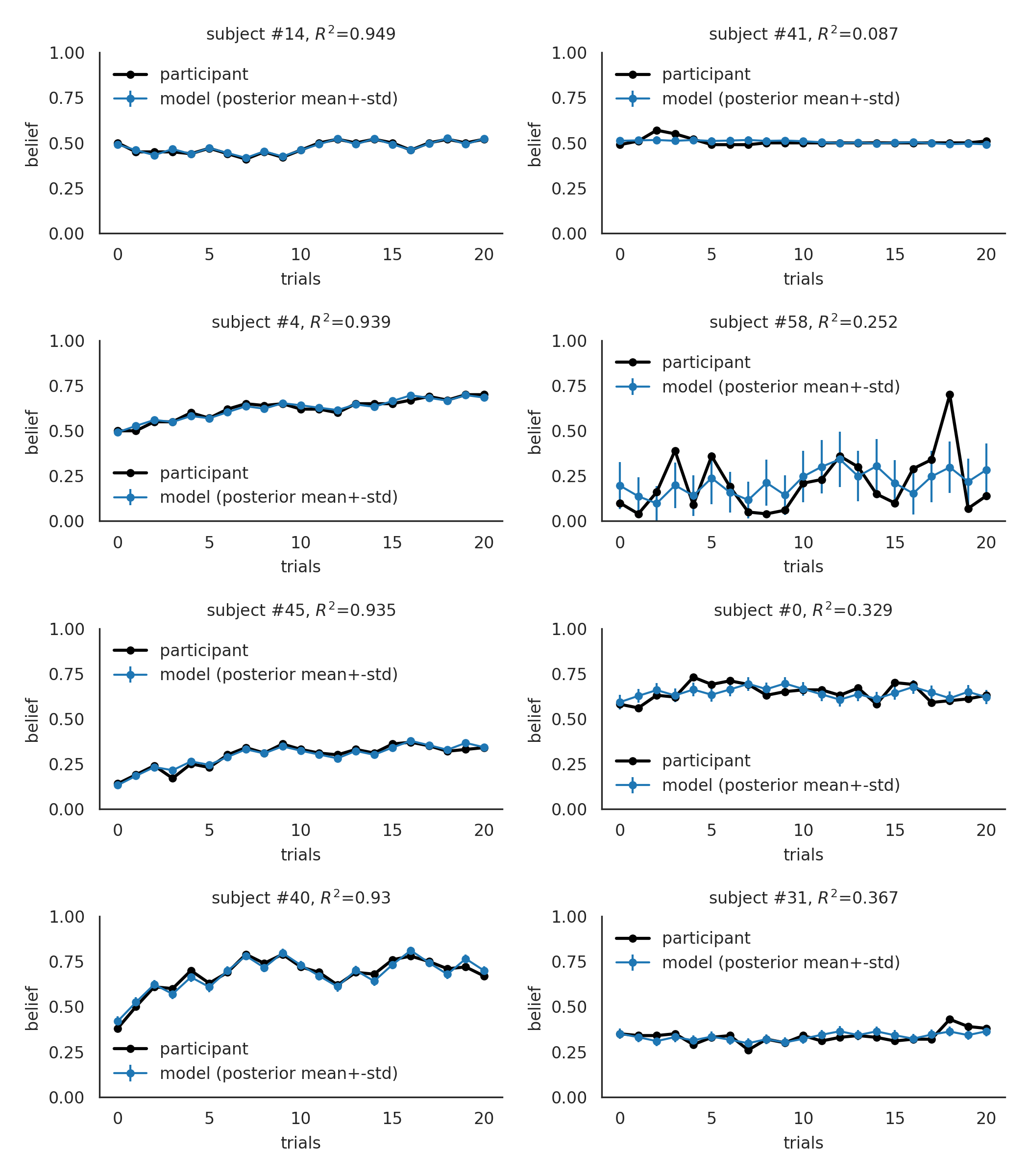

Supplement: S9 Fig — Participants in which model #3 fits best (left column) and worst (right column) are shown. The black line is the participant’s reported belief for self-referential judgments and the blue line is the model #3’s posterior mean prediction; error bars represent ±1 posterior standard deviation. The left columns shows that the model can fit extremely well (R-squared >0.93) for a variety of different of behaviors. The right column shows examples of behavior that cannot be captured, such as early updating followed by no updating (e.g., participant #41) or participants with highly variable magnitudes in updating (participant #58). Note that these two participants are not better fit by the second best model (model #10), with lower R-squared in all four cases. (TIF) [file pcbi.1010176.s015.tif]
